# AK2 deficiency compromises the mitochondrial energy metabolism required for differentiation of human neutrophil and lymphoid lineages

**DOI:** 10.1038/cddis.2015.211

**Published:** 2015-08-13

**Authors:** E Six, C Lagresle-Peyrou, S Susini, C De Chappedelaine, N Sigrist, H Sadek, M Chouteau, N Cagnard, M Fontenay, O Hermine, C Chomienne, P Reynier, A Fischer, I André-Schmutz, N Gueguen, M Cavazzana

**Affiliations:** 1 Laboratory of Human Lymphohematopoiesis, INSERM UMR 1163, Paris, France; 2 Paris Descartes–Sorbonne Paris Cité University, Imagine Institute, Paris, France; 3 SFR Necker, Plateforme de bioinformatique, Paris Descartes–Sorbonne Paris Cité University, Paris, France; 4 Department of Immunology and Hematology, Cochin Institute, INSERM U1016, Paris, France; 5 Department of Adult Hematology, Necker Children's Hospital, Assistance Publique-Hôpitaux de Paris, Paris, France; 6 Service de Biologie Cellulaire, Hopital Saint-Louis, Assistance Publique-Hôpitaux de Paris, Paris, France; 7 INSERM UMRS 940, Diderot - Sorbonne Paris Cité University, Paris, France; 8 UMR CNRS6214, INSERM 1083, Département de Biochimie et Génétique, Centre Hospitalier Universitaire, Angers, France; 9 Pediatric Immunology-Hematology and Rhumatology Department, Necker Children's Hospital, Assistance Publique-Hôpitaux de Paris, Paris, France; 10 Collège de France, Paris, France; 11 Biotherapy Department, Necker Children's Hospital, Assistance Publique-Hôpitaux de Paris, Paris, France; 12 Biotherapy Clinical Investigation Center, Groupe Hospitalier Universitaire Ouest, Assistance Publique-Hôpitaux de Paris, INSERM, Paris, France

## Abstract

Reticular dysgenesis is a human severe combined immunodeficiency that is primarily characterized by profound neutropenia and lymphopenia. The condition is caused by mutations in the adenylate kinase 2 (AK2) gene, resulting in the loss of mitochondrial AK2 protein expression. AK2 regulates the homeostasis of mitochondrial adenine nucleotides (ADP, ATP and AMP) by catalyzing the transfer of high-energy phosphate. Our present results demonstrate that AK2-knocked-down progenitor cells have poor proliferative and survival capacities and are blocked in their differentiation toward lymphoid and granulocyte lineages. We also observed that AK2 deficiency impaired mitochondrial function in general and oxidative phosphorylation in particular – showing that AK2 is critical in the control of energy metabolism. Loss of AK2 disrupts this regulation and leads to a profound block in lymphoid and myeloid cell differentiation.

Severe combined immunodeficiencies are inherited disorders characterized by a block in the differentiation of the T lymphoid lineage and defects in other hematopoietic lineages.^1, 2^ The autosomal recessive severe combined immunodeficiency known as reticular dysgenesis (RD) is characterized by the absence of neutrophils, T and natural killer (NK) lymphocytes and by bilateral sensorineural deafness. Red blood cells, platelets and/or B cells are affected in some cases. Clinical manifestations appear in the first few weeks after birth, owing to profound neutropenia that cannot be corrected by administration of granulocyte colony-stimulating factor (G-CSF). At present, the only available treatment for RD is hematopoietic stem cell transplantation.^3^

RD is known to be caused by mutations in the *AK2* gene, leading to an absence of AK2 protein expression.^4, 5^ AK2 belongs to the adenylate kinase family and is widely expressed in many tissues and in all hematopoietic cells. The AK2 protein is located in the intermembrane space of mitochondria, whereas other members of the AK family are cytoplasmic (AK1, 5, 7 and 8), nuclear (AK6) or located in the mitochondrial matrix (AK3 and AK4).^6, 7^ The AK2 protein regulates intracellular ATP levels by catalyzing the reversible transfer of a phosphate group in the reaction ATP+AMP ↔ 2 ADP.^6^ It is known that AK2 senses AMP, modulates metabolic signaling processes and maintains energy homeostasis in the cell. Recent studies have shown that the differentiation of hematopoietic stem cells (HSCs) requires high energy levels, which are provided by the activation of oxidative phosphorylation (OXPHOS) in the mitochondria.^8^ It has also been suggested that deregulation of AK2 function could be involved in the alteration of mitochondrial metabolism and, consequently, in the development of human disease.^9^

With a view to understand AK2's involvement in hematopoiesis, we developed an *in vitro* RNA interference strategy *via* lentiviral-mediated gene transfer of AK2 short hairpin RNAs into human hematopoietic progenitors or cell lines. Our present results demonstrate that in the absence of AK2 protein expression, progenitor cells could neither proliferate nor differentiate into lymphoid and granulocyte lineages. We also identified AK2 as a major regulator of energy metabolism – suggesting a direct link between the differentiation block observed in RD patients and the regulation of mitochondrial function.

## Results

### AK2 deficiency impairs survival and differentiation in T and NK lymphoid lineages

**Figure 1:**
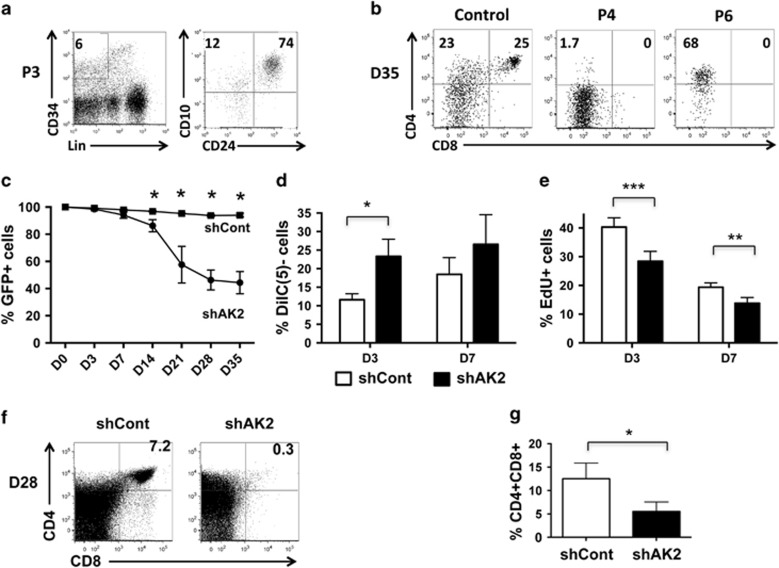
Impaired cell survival/proliferation and blockade of T-cell differentiation in AK2-deficient cells. (**a**) BM mononuclear cells from an RD patient (P3) were analyzed by flow cytometry, in order to evaluate the presence of the multilymphoid progenitor population (i.e., the CD10+CD24− population that accounts for 12% of the CD34+Lin− subset). (**b**) Two BM samples from RD patients (P4 and P6) and one sample from a healthy donor were sorted in order to purify CD34+ hematopoietic progenitors. To monitor T-cell differentiation, cells were seeded on OP9-hDelta1 cells. After 35 days of culture, we analyzed by flow cytometry for the presence of a CD4+CD8+ T-cell population. (**c**–**g**) CB CD34+ progenitors were transduced with shAK2 or shCont, sorted for GFP+ cells and plated on OP9-hDelta1 cells until D35. (**c**) The change over time in the percentage of GFP+ cells in shCont-transduced cells (black squares) or shAK2-transduced cells (black circles) (*P*=0.049, *P*=0.033, *P*=0.014 and *P*=0.012 at D14, D21, D28 and D35, respectively, *n*=4). (**d**) Mitochondrial membrane potential was analyzed in shCont-transduced cells (white bars) and shAK2-transduced cells (black bars) using the Mitoprobe DiIC(5) reagent 3 and then 7 days after the initiation of T-cell differentiation. The percentage of depolarization (corresponding to the proportion of viable 7AAD-negative cells that were negative for DiIC(5)) is shown (*P*=0.011 on D3 for shCont *versus* shAK2, *n*=6). (**e**) Cell proliferation was analyzed following incorporation of EdU 3 and then 7 days after the initiation of T-cell differentiation (*P*=0.0006 and *P*=0.008 on D3 and D7, respectively, *n*=6). (**f**) Flow cytometry analysis of GFP+ cells 28 days after the initiation of T-cell differentiation in the shCont and shAK2 conditions. (**g**) The proportion of DP CD4+CD8+ cells 28 days after initiation of T-cell differentiation in the shCont (white bars) and shAK2 conditions (black bars) (*P*=0.048); *P<0.05, **P<0.01, ***P<0.001

In order to monitor AK2's role in lymphoid T-cell differentiation, we took advantage of the availability of bone marrow (BM) samples from RD patients (referred as P3, P4 and P6 in our previous report^5^). These samples contained both Lin−CD34+CD10+CD24− and Lin−CD34+CD10+CD24+ progenitor populations – indicating that multilymphoid progenitors are not affected by AK2 deficiency (Figure 1a).

The cell differentiation potential of BM CD34+ progenitors was tested on OP9-hDelta1 stromal cells. On day (D) 35, we did not detect any double-positive (DP) CD4+CD8+ T cells among the AK2-deficient cells (in contrast to the control cells) (Figure 1b). It is noteworthy that for P6, we observed the emergence of a population of large CD4+ cells that very probably corresponded to monocytes.

In order to better characterize the role of AK2 during lymphoid differentiation, we looked at whether two independent shRNAs (shAK2 #1 and shAK2 #2) could knock down AK2 expression in CD34+ progenitors from cord blood (CB), relative to a control shRNA (shCont) (Supplementary Figure S1A). The transduced cells were sorted according to the green fluorescent protein (GFP) expression induced by the lentiviral constructs after culture on OP9-hDelta1 stromal cells. We followed the T-cell differentiation process in GFP+ cells by using the gating strategy described in Supplementary Figure S2. The two shRNAs revealed similar inhibition of proliferation and T-cell differentiation for shAK2 #1 and shAK2 #2 (Supplementary Figure S1B, S1C), although shAK2 #2 was slightly more potent as measured in an RT-qPCR analysis and in a T-cell differentiation assay. Further investigation of AK2's function was therefore performed with shAK2 #2. With AK2 knockdown, human GFP+CD34+ progenitors were poorly able to proliferate and survive during T-cell differentiation (Figure 1c). Between 3 and 7 days after the initiation of T-cell differentiation, we observed a twofold increase in mitochondrial membrane potential depolarization (Figure 1d) and a significant decrease in the percentage of proliferating cells (as evidenced by 5-ethynyl-2-deoxyuridine (EdU) uptake (Figure 1e)). The proportion and number of DP CD4+CD8+ T cells were much lower in the presence of shAK2 than in the presence of shCont – showing that the differentiation process *per se* was also affected (Figures 1f and g and Supplementary Figure S1C).

**Figure 2:**
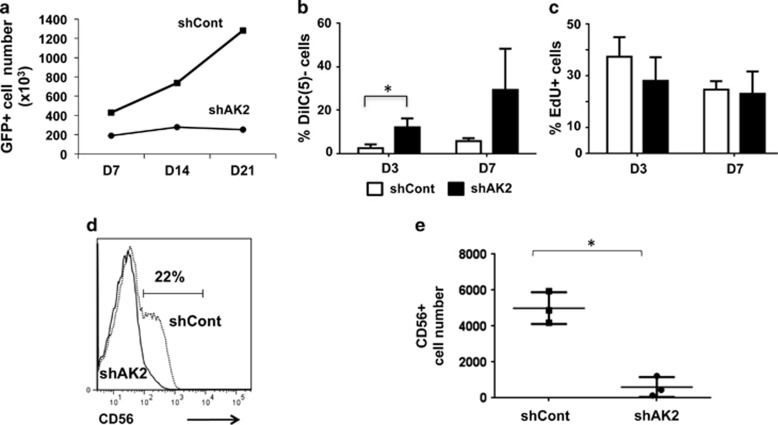
Impairment of survival and blockade of NK cell differentiation in AK2-deficient cells. CB CD34+ progenitors were transduced with shAK2 or shCont, sorted for GFP+ cells and seeded in NK cell differentiation medium for 21 days. (**a**) The change over time in the number of GFP+ cells during NK cell differentiation after transduction with shCont (black squares) or shAK2 (black circles). One of the three representative experiments is shown. (**b**) Mitochondrial depolarization was analyzed using the Mitoprobe DiIC(5) reagent 3 and then 7 days after initiation of NK cell differentiation in the shCont condition (white bars) and the shAK2 condition (black bars) (*P*=0.028 at D3, *n*=3). (**c**) Cell proliferation (incorporation of EdU) was analyzed 3 and 7 days after initiation of NK cell differentiation in the shCont condition (white bars) and the shAK2 condition (black bars) (*n*=3, *P*=not significant (NS)). (**d**) Flow cytometry analysis of GFP+ cells 21 days after initiation of NK cell differentiation in the shCont condition (dotted line) and the shAK2 condition (solid line). (**e**) The CD56+ cell number was evaluated 21 days after initiation of NK cell differentiation in the shCont (black squares) or shAK2 conditions (black circles) (*P*=0.014, *n*=3); *P<0.05

We used the same strategy to evaluate the ability of AK2-knocked-down CD34+ progenitors to differentiate into NK cells. We observed a significantly lower number of GFP+ cells in the shAK2 condition (relative to the shCont condition) (Figure 2a). Three days after the initiation of NK cell differentiation, greater disruption of the mitochondrial membrane potential was detected in shAK2 cells (Figure 2b). Proliferation did not appear to be lower in shAK2 cells (Figure 2c) than in shCont cells. On day 21, the number of CD56+ cells was significantly lower (Figures 2d and e) in the shAK2 condition (and with shAK2 #1, Supplementary Figure S1D) than in the shCont condition. These data demonstrated that NK cell differentiation was also dramatically affected by AK2 downregulation.

Taken as a whole, our data emphasize AK2's key role in the differentiation of hematopoietic cells towards T and NK lymphoid lineages.

### AK2 deficiency impairs the proliferation and differentiation of the granulocyte lineage

**Figure 3:**
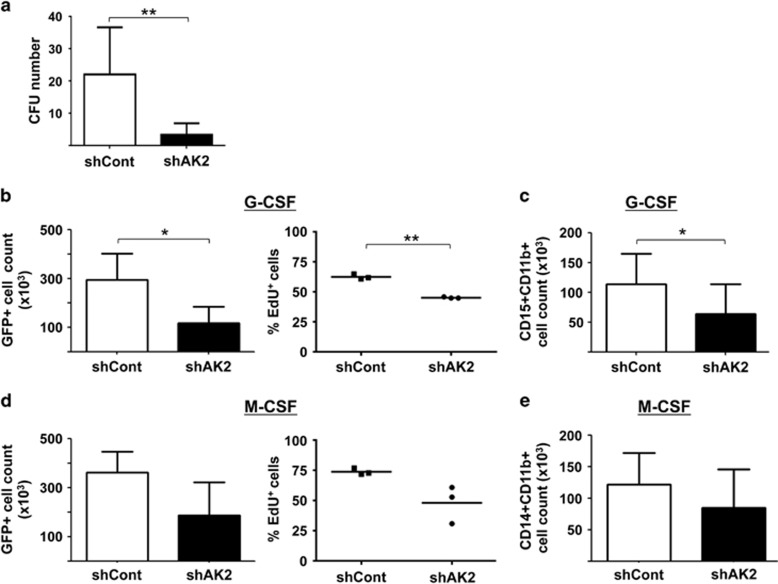
Impairment of granulocyte differentiation (but not monocyte differentiation) in AK2-deficient cells. CB CD34+ progenitors were transduced with shAK2 or shCont, sorted for GFP+ cells and seeded for 12 days in a methylcellulose culture (**a**) or for 14 days in a granulocyte culture with G-CSF or a monocyte culture with M-CSF (**b**–**e**). (**a**) The number of CFUs in a methylcellulose assay in the shCont condition (white bar) and shAK2 condition (black bar) (*P*=0.0062, *n*=6). The CFUs include both CFU-G and CFU-M colonies. (**b**) The total number of GFP+ cells in a G-CSF culture after transduction with shCont (white bar) or shAK2 (black bar) (left panel, *P*=0.026, *n*=3) and the proportion of EdU+ cells in shCont (black squares) or shAK2 (black circles) (right panel, *P*=0.0013, *n*=3). (**c**) The total number of CD15+CD11b+ granulocytes in a G-CSF culture after transduction with shCont (white bar) or shAK2 (black bar) (*P*=0.012, *n*=3). (**d**) The total number of GFP+ cells in an M-CSF culture after transduction with shCont (white bar) or shAK2 (black bar) (left panel) and the percentage of EdU+ cells in each condition (right panel, *P*=NS, *n*=3). (**e**) The total number of CD14+CD11b+ monocytes after an M-CSF culture after transduction with shCont (white bar) or shAK2 (black bar) (*P*=NS, *n*=3); *P<0.05, **P<0.01

In order to evaluate the impact of AK2 deficiency on myeloid differentiation, CD34+ progenitors transduced with the shAK2 or shCont lentiviral vector were sorted and the CD34+GFP+ cells were cultured either on methylcellulose or in the presence of G-CSF or macrophage colony-stimulating factor (M-CSF). In a colony-forming unit (CFU) assay, fewer CFUs were detected in the shAK2 condition than in the shCont condition – demonstrating the low clonogenic capacity of AK2-deficient progenitors (Figure 3a). Similar inhibition was observed with shAK2 #1 (Supplementary Figure S1E). These data also show that AK2 expression is required during the very first stages of hematopoietic differentiation.

To measure granulocyte differentiation, cells were cultured in suspension in the presence of G-CSF. As shown in Figure 3b, cell proliferation (as measured by the total GFP+ cell count and EdU uptake) was significantly lower in the shAK2 condition than in the shCont condition. Furthermore, we observed an inhibition of granulocyte differentiation, as evidenced by the low number of cells co-expressing CD15+CD11b+ markers on day 8 (Figure 3c). Conversely, differentiation of CD34+ cells towards the monocyte lineage was weakly (but not significantly) affected by AK2 knockdown (Figures 3d and e). The cells' phenotype closely mimics that of RD patients, who present profound neutropenia but normal monocyte counts. We conclude that AK2 has a key role in the proliferation, survival and differentiation process of the neutrophil lineage.

### Ectopic expression of the anti-apoptotic protein Bcl2 or adenylate kinase 1 does not compensate for AK2 knockdown

In view of the poor survival observed in AK2-deficient cells, we next looked at whether the anti-apoptotic regulator Bcl2 could restore cell survival. CB CD34+ progenitor cells were transduced with a Bcl2-GFP and a shAK2-mCherry lentiviral construct. After confirming Bcl2 expression and AK2 knockdown (Supplementary Figure S3A), we tested the ability of the sorted GFP+mCherry+ population to differentiate into T cells. After 42 days of culture, Bcl2 expression by the shAK2 progenitors was partially (but not significantly) associated with greater survival of the GFP+Cherry+ population (Supplementary Figure S3B) and the percentage of DP CD4+CD8+ cells (Supplementary Figure S3C). Indeed, Bcl2 overexpression did not bypass the T-cell differentiation blockade or compensate for the lack of AK2. Similarly, the low number of CFUs in the shAK2 condition (relative to the shCont condition) was not modified by Bcl-2 overexpression (Supplementary Figure S3D).

Using the same strategy, we tested the ability of adenylate kinase 1 (AK1, which is also involved in the regulation of intracellular ATP levels) to rescue the survival defect observed in AK2 knockdown (Supplementary Figure S4A). The results of both the CFU assay (Supplementary Figure S4B) and the NK cell differentiation assay (data not shown), confirmed that AK1 overexpression did not alleviate the differentiation blockade.

Taken as a whole, these results indicate that Bcl2 or AK1 proteins cannot compensate for AK2 deficiency during the differentiation of CD34+ cell towards lymphoid or myeloid lineages. Hence, AK2's function is specific and may be linked to other pathways regulating cell survival and/or metabolism.

### AK2 deficiency in the promyelocytic HL60 cell line blocks granulocyte differentiation and induces mitochondrial respiration defects

In order to further understand AK2's mechanism of action, and because very few cells can be harvested from knockdown experiments on CD34 progenitors, we took advantage of the properties of the promyelocytic HL60 cell line. The latter is able to differentiate towards a neutrophil lineage (upon treatment with all-trans retinoic acid) or a monocyte lineage (upon treatment with vitamin D3).

**Figure 4:**
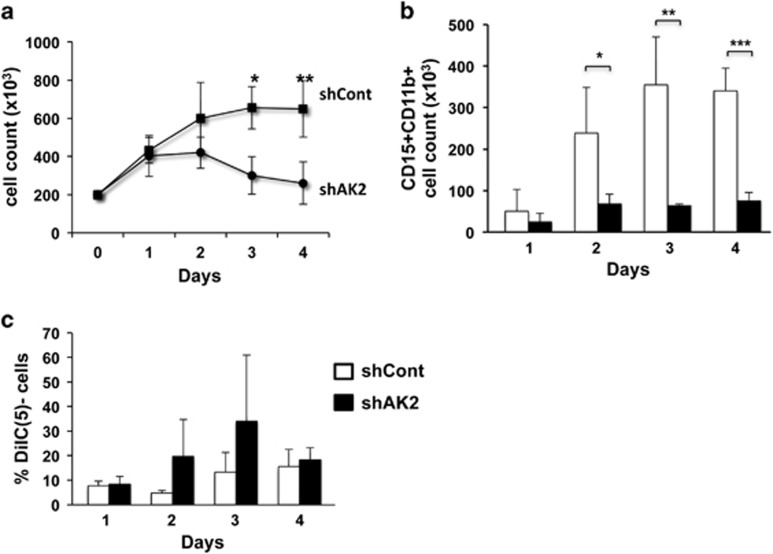
Impaired survival and blockade of neutrophil cell differentiation in the AK2-deficient HL60 cell line. The promyelocytic cell line HL60 was transduced with shAK2 or shCont, selected with puromycin and seeded in culture with 10 *μ*M ATRA for 4 days, in order to induce neutrophil differentiation. (**a**) The change over time in total cell count during neutrophil differentiation in shCont (black squares) or shAK2 (black circles) (*P*=0.013 on D3 and *P*=0.002 on D4, *n*=4). (**b**) The CD15+CD11b+ neutrophil count in differentiated HL60 cells transduced with shCont (white bar) or shAK2 (black bar) (*P*=0.033 on D2, *P*=0.008 on D3 and *P*=0.0003 on D4; *n*=4). (**c**) Mitochondrial depolarization was analyzed using Mitoprobe DiIC(5) reagent in HL60 cells transduced with shCont (white bar) and shAK2 (black bar) (*n*=4, *P*=NS); *P<0.05, **P<0.01, ***P<0.001

Our analysis of neutrophil differentiation following AK2 knockdown in the HL60 cell line revealed a drastic inhibition of cell proliferation (Figure 4a) and a significant inhibition of differentiation, as shown by the low number of mature granulocytes obtained after 4 days of culture (Figure 4b). We observed slightly greater depolarization of the mitochondrial membrane depolarization in AK2-deficient cells than in controls (Figure 4c), although the difference was not statistically significant. In contrast to neutrophil differentiation, monocyte differentiation was not affected by AK2 knockdown (Supplementary Figure S5). The impact of AK2 downregulation in the HL60 cell line fits well with the data reported for primary cells during myeloid differentiation. Accordingly, this is a valid model for better understanding AK2's function during the differentiation process and, in particular, establishing whether the absence of AK2 can deregulate mitochondrial metabolism.

**Figure 5:**
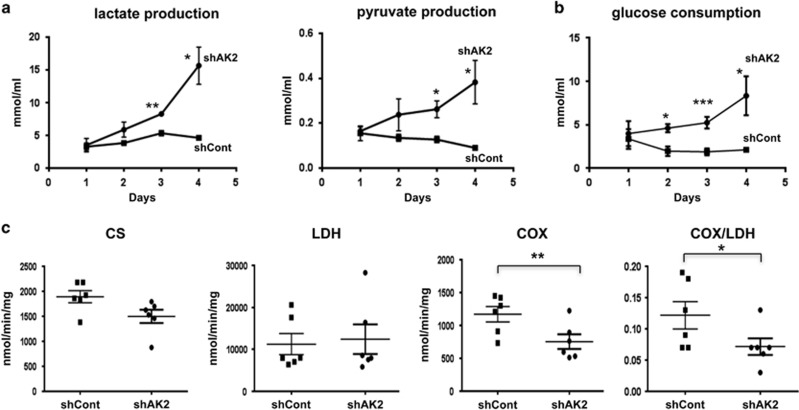
AK2 deficiency compromises energy metabolism during neutrophil differentiation. HL60 cells were transduced with shAK2 or shCont, selected with puromycin and seeded in culture with 10 *μ*M ATRA for 4 days, in order to induce neutrophil differentiation. (**a**) Lactate and pyruvate levels in the cell culture supernatant of cells transduced with shCont (black squares) or shAK2 (black circles). There were significant differences in lactate (*P*= 0.0013 and 0.014 at D3 and D4, respectively) and pyruvate levels (*P*= 0.013 and 0.020 at D3 and D4, *n*=4) when comparing the two conditions. (**b**) Glucose consumption by cells transduced with shCont (black squares) or shAK2 (black circles) was calculated by measuring the difference in glucose concentration in the cell medium between the initial concentration and each time point. There were significant differences on D2, D3 and D4 (*P*=0.024, 0.0006 and 0.035, respectively, *n*=4). (**c**) Mitochondria function, as evidenced by the activities of CS, LDH and COX complex IV on D4 of culture in HL60 cells transduced with shCont (black squares) and shAK2 (black circles) (*P*=NS for CS and LDH, *P*=0.009 for COX and *P*=0.04 for COX/LDH, *n*=6); *P<0.05, **P<0.01, ***P<0.001

During HL60 neutrophil differentiation, we first evaluated cell metabolism by measuring lactate and pyruvate production and glucose consumption in the culture supernatant. In the absence of AK2 (i.e., the shAK2 condition), significant accumulation of lactate and pyruvate was observed after 3 days of culture (relative to the control condition) (Figure 5a). The enhancement of glycolysis in AK2-deficient cells was confirmed by a statistically significant elevation in glucose consumption (relative to the control condition) from 2 days of culture onwards (Figure 5b). We also measured the activities of citrate synthase (CS, the first enzyme in the tricarboxylic acid cycle, used as a marker of the mitochondrial mass), lactate dehydrogenase (LDH, which catalyzes the conversion of pyruvate to lactate in the cytoplasm) and cytochrome oxidase (COX, also known as complex IV – the final acceptor in the respiratory chain). After 4 days of culture (Figure 5c), the CS and LDH activities in the shAK2 and control conditions were similar. However, the COX activity in the shAK2 condition was half that observed in the shCont condition. Although glucose consumption is elevated in AK2-deficient cells, mitochondrial activity is associated with incomplete oxidation and the accumulation of pyruvate and lactate.

**Figure 6:**
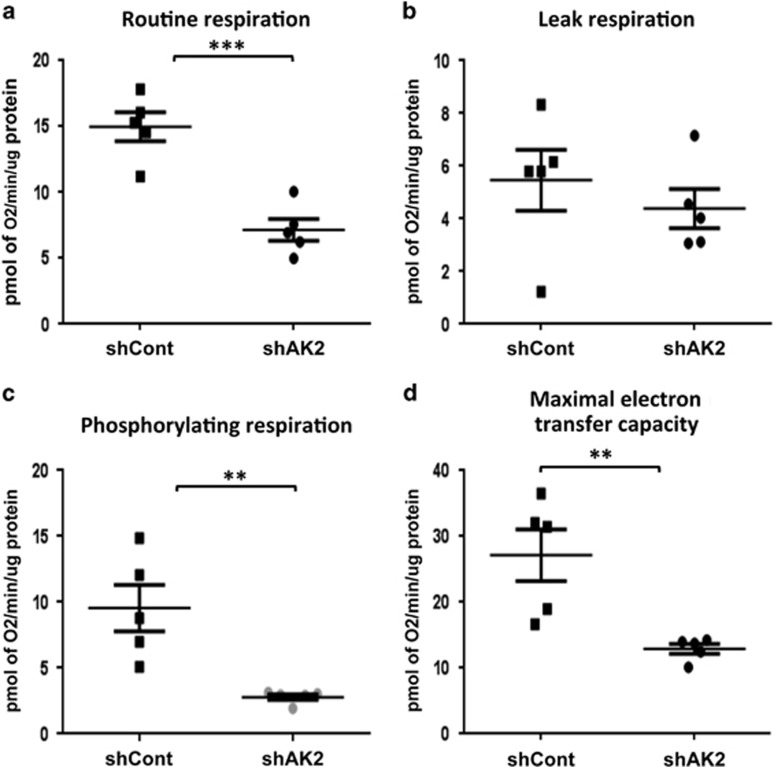
AK2 is required for mitochondrial respiration. HL60 cells were transduced with shAK2 or shCont, selected with puromycin and seeded in culture with 10 *μ*M ATRA, to induce neutrophil differentiation. After 4 days of culture, oxygen consumption was compared in shCont (black square) and shAK2 (black circle) conditions. (**a**) Routine respiration (**R**, *P*=0.0007), (**b**) oligomycin-insensitive proton leak respiration (**O**, *P*=NS), (**c**) phosphorylating respiration (driving ATP synthesis) (**R-O**, *P*=0.0071) and (**d**) maximum electron transfer capacity (assessed using the uncoupler FCCP (*P*=0.0076)); *P<0.05, **P<0.01, ***P<0.001

These results suggested that mitochondrial function is altered in the absence of AK2 expression. To further explore this modification, we assessed mitochondrial respiration by measuring oxygen consumption rates in shAK2- and shCont-HL60 cell lines treated with all-trans retinoic acid. We observed that AK2 downregulation is associated with a significant decrease in routine respiration (Figure 6a) but not a change in mitochondrial proton leak, as reflected by the oligomycin-insensitive respiration rates (Figure 6b). The proportion of respiratory activity coupled to ATP production (i.e., the rate of phosphorylating respiration) can be estimated by subtracting the leak respiration from the routine respiration. As shown in Figure 6c, AK2 inhibition clearly impaired phosphorylating respiration. Lastly, the maximum electron transfer capacity (obtained by uncoupling electron transport from oxidative phosphorylation by adding the mitochondrial uncoupler carbonyl cyanide p-trifluoromethoxyphenylhydrazone (FCCP)) was also significantly lower in the shAK2 condition than in the control condition (Figure 6d) and might be a limiting factor for efficient ATP synthesis. Therefore, the absence of AK2 significantly impairs the respiratory chain's activity.

Taken as a whole, these results demonstrate that AK2 is a key factor for mitochondrial function in general and respiratory chain activity in particular.

### The mRNA expression profile of AK2-deficient cells

In order to further characterize the molecular pathways regulated by AK2, we analyzed the transcriptome of AK2-deficient cells during the early stages of the T-cell differentiation process (on D3). To rule out off-target effects of shRNA, we investigated the molecular signature of cells transduced with either shAK2 #1 or shAK2 #2.

**Figure 7:**
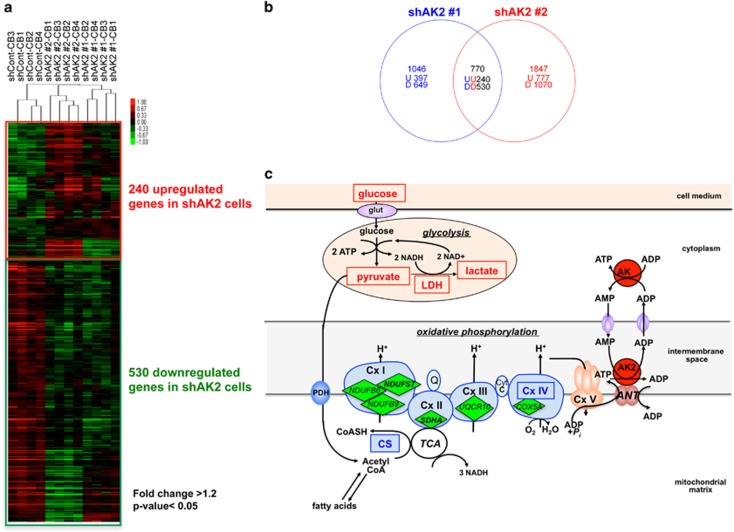
Gene expression profiling of AK2-deficient cells, showing alterations in mitochondrial metabolism. (**a**) A heat map corresponding to the 770 genes deregulated in the shAK2 #1 and shAK2 #2 conditions, relative to the shCont condition. The hierarchical clustering of the four CB (CB1 to 4) replicates is shown. Significant differences are based on a 1.2-fold difference and a *P*-value<0.05. (**b**) A Venn diagram comparing upregulated (U) and downregulated (D) genes in the shAK2 #1 (blue) and shAK2 #2 (red) conditions, relative to the shCont condition. (**c**) A schematic model of AK2's function with respect to oxidative and glycolytic metabolism. Metabolites and enzymes measured in our assays in Figure 5 are framed. The respiratory chain protein genes found to be downregulated in shAK2 condition are shown in green

A supervised clustering approach identified 240 upregulated genes and 530 downregulated genes (fold-change >1.2; *P*<0.05) when comparing shAK2 #1 and shAK2 #2 with shCont (Figures 7a and b; Supplementary Table S1). In a gene set enrichment analysis, we found that many of the upregulated and downregulated genes were involved in the cell cycle and metabolism (Table 1; false-discovery rate q-value <0.1). The observed upregulation of CDKN1C (also known as p57^KIP2^) and downregulation of CCNE1, FOXM1 and several genes involved in the control of chromosomal replication (CDC6, CDC45, CHEK2, MCM2, MCM3, MCM7) (Supplementary Table S1) suggest that during the differentiation process, the cell cycle is significantly altered in shAK2-transduced cells.

The results of our gene expression analysis also emphasized the impairment of metabolic pathways associated with mitochondrial functions in the absence of AK2: OXPHOS (including assembly factors for each of the respiratory chain complexes), the tricarboxylic acid cycle, pyruvate dehydrogenase, mitochondrial translation, the mitochondrial import of proteins and metabolites, and fatty acid metabolism (Supplementary Table S2). Moreover, the observed downregulation of COX5A (one of the subunits of complex IV) (Figure 7c) agreed with the complex's low functional activity in our experiments on HL60 cells.

It is noteworthy that some of the up/downregulated genes were involved in the nucleotide metabolism required for genome maintenance, replication and expression (Supplementary Table S2).

In conclusion, the gene set enrichment analysis results confirmed that AK2 deficiency is associated with a general imbalance of mitochondrial metabolism. The latter is probably responsible for the impaired survival, proliferation and differentiation of AK2-deficient hematopoietic cells.

## Discussion

Our present results demonstrate that AK2 has a specific, critical role in mitochondrial metabolism in general and oxidative phosphorylation in particular. A lack of AK2 inhibits lymphoid and neutrophil differentiation. To mimic the AK2 deficiency in RD, we knocked down AK2 expression in CB CD34+ progenitor cells. In the absence of AK2 expression, we found that hematopoietic progenitors could no longer differentiate along lymphoid or granulocyte lineages. In addition to this differentiation block, we observed a negative effect of AK2 knockdown on cell survival and proliferation in the context of differentiation. This effect was correlated with the dysregulated expression of cell cycle genes, including upregulation of CDKN1C (a negative regulator of cell proliferation). These findings are in agreement with a report that AK2 regulates viability and cell growth during larval development in *Drosophila melanogaster*.^10^ It is important to note that AK2 knockdown did not alter the number of hematopoietic progenitors or the latter's ability to proliferate (Figure 1a and Supplementary Figure S6); in fact, AK2 is required when the cells commit to a specific lineage pathway.

It has been reported that in mature human cell lines, AK2 mediates mitochondrial apoptosis by binding to Fas-associated with death domain protein (FADD) and caspase-10.^11, 12^ The latter observation does not fit with our present data and the phenotype of RD.^4, 5^ Moreover, we found that AK2 knockdown in murine hematopoietic progenitors also impairs T-cell and neutrophil differentiation (data not shown). As mice do not express the caspase-10 gene,^13^ our observation suggests that other pathways are involved in AK2's function. We also demonstrated that expression of the anti-apoptotic factor Bcl2 was not enough to reverse the survival and differentiation defects in AK2 knockdown cells (Supplementary Figures S3 and S7). Taken as a whole, these results suggest that cell death is not an intrinsic effect of AK2 deficiency but rather a consequence of the alteration of AK2 signaling pathway.

Given that AK2's main reported function is related to energy transfer (regulation of ATP flux), we looked at whether AK1 (another member of the adenylate kinase family which is not expressed in hematopoietic lineages other than erythrocytes^4^) could compensate for AK2 deficiency. We found that AK1 could not restore lymphoid or granulocyte differentiation of AK2-knocked-down CD34+ cells. The cytoplasmic location of AK1 probably explains why the latter protein could not compensate for mitochondrial AK2 deficiency.

To study the relationship between AK2 and mitochondrial metabolism, we performed several experiments with the HL60 cell line. In AK2-deficient cells, we observed an impairment of oxidative metabolism (low COX activity and low COX/LDH ratio), which was partially compensated by an elevated rate of glycolysis (i.e., increased lactate production) relative to the control condition. These metabolic changes are in accordance with a previous report on tumor cell lines, in which AK2 knockdown was associated with reduced proliferation, increased glucose uptake and increased lactate production.^14^ Moreover, it is noteworthy that CS activity was not affected by AK2 knockdown – suggesting that the absence of AK2 expression has no effect on the number of mitochondria but did influence mitochondrial activity. Electron microscopy experiments in this HL60 cell model did not reveal any alterations in the mitochondria ultrastructure (Supplementary Figure S8).

On the basis of oxygraphy experiments, we confirmed that AK2 is a key factor for mitochondrial respiration because phosphorylating respiration (coupled to ATP synthesis) is abnormally low in AK2-deficient cells. This limitation of basal oxidative activity probably explains why AK2-deficient cells could not respond to the high energy demand required for differentiation.

Our present data are reminiscent of the ‘metabolic switch' observed when HSCs begin to differentiate. In endosteal BM niches, the immature HSCs are in a quiescent state and their low energy needs are fully met by glycolysis. Following exposure to various differentiation stimuli, HSCs undergo a metabolic switch toward mitochondrial OXPHOS in order to satisfy a greater energy demand.^15^ The low ATP level observed in HSCs (relative to their lineage-positive counterpart) fits with this observation.^16^

Another recent report illustrated the critical link between the regulation of mitochondrial respiration and hematopoietic differentiation. Yu *et al.*^17^ demonstrated that in PTEN–like mitochondrial phosphatase (PTPMT1, located in the inner mitochondrial membrane) conditional knock-out mice, low oxidative capacity abrogates the differentiation of HSCs towards myeloid and lymphoid lineages. Of note, the low oxidative capacity of PTPMT1-deficient cells was not associated with a detectable change in the ATP level.

Our transcriptome analysis confirmed the alteration in OXPHOS, because several genes involved in human OXPHOS disorders (e.g., Leigh syndrome)^18^ were downregulated in AK2-deficient cells. Interestingly, microarray analyses in *Drosophila* have demonstrated that AK2 defect is associated with deafness and impaired mitochondrial function.^19^ The bilateral deafness found in RD patients might thus also result from an impairment in mitochondrial metabolism. This hypothesis is supported by our present data, especially by our transcriptome analysis as inherited deafness genes (such as COCH (coding for cochlin), TIMM8B, PRPS1 and tRNA synthetase) are downregulated in AK2-deficient cells.^20, 21, 22, 23^

Taken as a whole, these data demonstrate that AK2 regulates mitochondrial activity and thus influences lymphoid and granulocyte differentiation. However, our data do not explain why the AK2 deficiency in RD patients does not alter the monocyte compartment. Hence, a redundant pathway might provide the energy required for monocyte differentiation. The latter hypothesis is supported by a recent report that CKMT1 (a mitochondrial creatine kinase involved in ATP flux) is expressed after commitment to monocyte differentiation (but not granulocyte differentiation) in AK2-knocked-down HL60 cells.^24^ Hence, CKMT1 might compensate for the lack of AK2 in the monocyte lineage.

Interestingly, AK2's mechanism of action herein described (linking mitochondria and the control of cell fate) resembles the role of adenylate kinase in stem cell differentiation in other tissue (such as the heart, where AK deficiency disrupts cardiogenesis by compromising the mitochondrial network^25^).

Taken as a whole, our results suggest that the AK2 signaling pathway is associated with the regulation of mitochondrial metabolism. In the absence of AK2 expression, the export of high-energy phosphate compounds from the mitochondrion is impaired. Energy failure and nucleotide disequilibrium affect cell survival, proliferation and differentiation, which in turn impair differentiation towards neutrophil and lymphoid lineages. Hence, our results highlight the close relationship between energy balance and cell differentiation and thus show how the impairment of mitochondrial metabolism can result in one of the most profound immunodeficiency syndromes yet characterized.

## Materials and Methods

### Cell preparations

CB samples eligible for research purposes (from the Cord Blood Bank at Saint Louis Hospital, Paris, France) and BM mononuclear cells from controls and patients were harvested following the provision of written, informed consent. Control BM cells corresponded to the unused residue of an allogeneic hematopoietic stem cell harvest from a healthy adult donor. Patient BM cells came from residual BM after myelogram analysis as part of the diagnostic procedure. The constitution of a collection of BM samples had been approved by the regional investigational review board and the French Ministry of Research (references: DC 2011-1338 and DC 2011-1421). The collection was used in accordance with French legislation and the ethical tenets of the Declaration of Helsinki. Mononuclear cells were isolated by density separation on Lymphoprep (Axis-Shield, Oslo, Norway). CD34+ hematopoietic progenitors were sorted magnetically using the autoMACSpro separator (Miltenyi Biotec, Bergisch Gladbach, Germany). The purity of the CD34+ cells was checked with a MACSQuant analyzer (Miltenyi Biotec).

### Cell lines

The HL60 human promyelocytic leukemia cell line was maintained in RPMI-1640 medium supplemented with 10% fetal bovine serum. Neutrophil or monocyte differentiation was induced by treating the HL60 cells with 10 *μ*M all-trans retinoic acid or 100 nM vitamin D3, respectively (Sigma Aldrich, St Louis, MO, USA). The OP9-hDelta1 cell line was generated as described previously^26^ and co-expressed the human Notch ligand Delta1 (hDelta1) and GFP.

### Flow cytometry analysis and cell sorting

Monoclonal antibodies against CD4 (OKT4), CD7 (M-T701), CD8 (RPA-T8), CD10 (97C5), CD11b (D12), CD14 (M5E2), CD24 (ML5), CD34 (8G12), CD56 (B159), mouse IgM, mouse IgG1k, mouse IgG2a, mouse IgG2b isotype controls, annexin V and 7-aminoactinomycin D (7AAD) were obtained from BD Biosciences (San José, CA, USA). CD15 (80H5) and mouse IgM control antibodies were purchased from Beckman Coulter (San Diego, CA, USA). Murine *β*1-integrin (CD29, HM*β*1-1) was from Biolegend (San Diego, CA, USA). Sytox Blue was obtained from Life Technologies (Carlsbad, CA, USA). The lineage cocktail has been described elsewhere.^27^ After staining, cells were analyzed on a MACSQuant analyzer, with gating on viable, 7AAD-negative or Sytox Blue-negative cells. The data were processed using FlowJo software (Treestar, Ashland, OR, USA).

### Mitotracker DiIC(5) staining and cell cycle analysis

The mitochondrial membrane potential was measured using DiIC(5) staining, according to the manufacturer's instructions. Treatment with CCCP was used as a positive control for depolarization (Life Technologies). Cell cycle analyses were performed by measuring the incorporation of the nucleoside analogue EdU into DNA (*via* a 6-h incubation with 10 *μ*M EdU) according to the manufacturer's instructions (using the Click-iT EdU kit from Life Technologies).

### Construction vectors

The shRNAs were cloned into the *Age*I and *Eco*RI sites of a pLKO.1 lentiviral vector (Addgene, Cambridge, MA, USA); the target sequences for shAK2 #1, shAK2 #2 and shCont are available in the Supplementary material. We generated pLKO-GFP variants of these vectors by replacing the puromycin resistance cassette (*Bam*HI and *Kpn*I sites) by the GFP reporter. Lentiviral supernatants were produced by the vector facility at SFR BioSciences Gerland-Lyon Sud (Lyon, France). All procedures with genetically modified cells have been approved by the French National Biotechnology Council and the French Ministry of Research (reference: 6304/4).

### Transduction of human CD34+ progenitor cells and HL60 cells

CB CD34+ cells were cultured overnight as previously described^5^ and then underwent two rounds of transduction with the appropriate lentiviral supernatant at a multiplicity of infection of 100. After 2 days of culture, the GFP+ cells were sorted on a FACSAria machine (BD Biosciences) prior to *in vitro* differentiation. For transduction of the HL60 cell line, we used the native pLKO.1 vector containing the puromycin resistance gene. The cells were transduced twice with lentiviral supernatant in the presence of polybrene (4 *μ*g/ml, Sigma Aldrich). To select cells expressing the transgene, the culture medium was supplemented with puromycin (4 *μ*g/ml, Invivogen, San Diego, CA, USA) for at least 5 days.

### *In vitro* differentiation assays

The CD34+ cells' ability to form CFUs was evaluated in a clonal assay on methylcellulose (MethoCult H4535, Stemcell Technologies, Vancouver, BC, Canada). The differentiation of CD34+ cells into granulocytes or monocytes was evaluated after an 8-day culture in X-VIVO-20 medium (Lonza, Switzerland) supplemented with 10% fetal bovine serum (Life Technologies), human stem cell factor (100 ng/ml) and human G-CSF (100 ng/ml) or human M-CSF (100 ng/ml). The CD34+ cells' ability to differentiate along the NK lineage was evaluated after a 21-day culture in X-VIVO-20 supplemented with fetal bovine serum, human stem cell factor and human interleukin-15 (20 ng/ml). Lastly, the CD34+ cells' ability to differentiate along the T-cell lineage was measured using coculture with OP9-hDelta1 stromal cells, as described elsewhere.^26^ All cytokines were purchased from Peprotech (Rocky Hill, NJ, USA).

### Measurement of metabolite levels and enzyme activity

Glucose and lactate concentrations in the culture media were determined spectrophotometrically using a glucose assay kit and a lactate assay kit, respectively (Abbott, Rungis, France). The pyruvate concentration was measured using a pyruvate assay kit (DiaSys, Condom, France), according to the manufacturer's instructions. The activities of respiratory chain complex IV, CS and LDH in HL60 cell lines were measured as described elsewhere.^28, 29^ The oxygen consumption rate was determined at 37°C using high-resolution respirometry with an Oroboros O2K oxygraph (Innsbruck, Austria) according to the method used by Hutter *et al.*^29^ The analysis started with the measurement of routine respiration, which is defined as cellular respiration in the culture medium (DMEM-F12, 1 mM pyruvate) in the absence of additional substrates or effectors. Next, the mitochondrial ATP synthesis was inhibited using oligomycin (2 *μ*g/ml) and oxidative phosphorylation was uncoupled by stepwise titration with the uncoupler FCCP (up to optimum concentrations in the range 0.8–1.2 *μ*M). Lastly, the specificity of mitochondrial respiration was checked by adding the complex III inhibitor antimycin A (2 *μ*g/ml). For all the various parameters, data were normalized against the protein levels in the oxygraph chamber. Routine respiration was defined as baseline respiration without treatment. Leak respiration was defined as the respiration measured in the presence of oligomycin (an ATP synthase inhibitor). Phosphorylating respiration was defined as routine respiration minus oligomycin-insensitive respiration. Maximum electron transfer capacity was defined as the maximum respiratory capacity in the presence of FCCP.

### Transcriptome analysis

Total RNA was isolated from sorted GFP+ progenitors after 3 days of culture on OP9-hDelta1 cells, using the RNeasy Plus Micro kit (Qiagen, Dusseldorf, Germany). Two-round amplification was performed with the ExpressArt C&E mRNA amplification nano kit (AmpTec, Hamburg, Germany). Microarray experiments were performed on Human Genome 433 plus 2.0 GeneChips, according to standard Affymetrix protocols. The arrays were read with a confocal laser (GeneChip Scanner 3000 7G, Affymetrix, Santa Clara, CA, USA). The CEL files were generated using GeneChip Command Console (AGCC) software (Affymetrix). Gene expression levels were normalized using the GC-RMA algorithm and flags were computed using MAS5 algorithm. Quality assessment of the chips was performed with an affyQCReport R package (Affymetrix). Intergroup comparisons were performed using a paired Student's *t* test. Cluster analysis was performed by hierarchical clustering using the Spearman correlation similarity measure and the average linkage algorithm. Gene set enrichment analysis was performed with gene set enrichment analysis software using the Reactome pathways derived from the Molecular Signatures Database. The microarray data have been deposited at ArrayExpress (EBI) with the accession number E-MTAB-2680.

### Statistical analysis

For all analyses, three or more independent experiments were performed. Data are reported as the mean±standard error of the mean (S.E.M.). A one-tailed, paired *t* test was performed using Prism 4 software (GraphPad, La Jolla, CA, USA). The threshold for statistical significance was set to *P*<0.05.

## Figures and Tables

**Table 1:** Gene sets enriched in shAK2 expressing hematopoietic progenitors

**Gene sets**	**NES**	**FDR q-val**
*Cell cycle control*		
DNA strand elongation	−1.88	0.006
Mitotic M M G1 phases	−1.84	0.01
Activation of the pre-replicative complex	−1.79	0.022
Activation of the ATR response to replication stress	−1.77	0.028
Cell cycle mitotic	−1.76	0.023
G2 M checkpoints	−1.76	0.023
DNA replication	−1.7	0.033
Mitotic prometaphase	−1.69	0.032
Cell cycle checkpoints	−1.67	0.04
M G1 transition	−1.66	0.045
Cell cycle	−1.65	0.05
Lagging strand synthesis	−1.64	0.047
Extension of telomeres	−1.62	0.059
		
*Signaling pathways*		
Signaling by BMP	1.85	0.039
		
*Metabolism*		
Nitric oxide stimulates guanylate cyclase	1.78	0.057
Metabolism of steroid hormones and vitamins A and D	−1.75	0.023
Mitochondrial protein import	−1.69	0.035
Glucose metabolism	−1.6	0.068
Respiratory electron transport ATP synthesis	−1.6	0.068
		
*Others*		
Kinesins	−1.74	0.021
Steroid hormones	−1.72	0.028
Amine-derived hormones	−1.72	0.026
mRNA processing	−1.67	0.043
DNA repair	−1.64	0.051
MHC class II antigen presentation	−1.64	0.05
Processing of capped intron containing pre mRNA	−1.64	0.048
Amine compound SLC transporters	−1.62	0.057
Amino acid transport across the plasma membrane	−1.62	0.058

Abbreviations: FDR: false discovery rate; NES, normalized enrichment score.

Gene set enrichment analysis with the REACTOME gene sets from the Molecular Signatures database

## Abbreviations

7-AAD: 7-Aminoactinomycin D
AK2: adenylate kinase 2
ATRA: all-trans retinoic acid
BM: bone marrow
CFU: colony-forming unit
CS: citrate synthase
COX: cytochrome oxidase
DP: double-positive
EdU: 5-ethynyl-2-deoxyuridine
FCCP: carbonyl cyanide p-trifluoromethoxyphenylhydrazone
G-CSF: granulocyte colony-stimulating factor
GFP: green fluorescent protein
HSC: hematopoietic stem cell
LDH: lactate dehydrogenase
M-CSF: macrophage colony-stimulating factor
NK: natural killer
OXPHOS: oxidative phosphorylation
RD: reticular dysgenesis
