# Supplementary material for: AK2 deficiency compromises the mitochondrial energy metabolism required for differentiation of human neutrophil and lymphoid lineages
Source: Cell Death Dis. 2015 Aug 13;6(8):e1856–. doi: 10.1038/cddis.2015.211 (PMC4558504; doi:10.1038/cddis.2015.211)
Supplement: Supplementary Information [file cddis2015211x1.pdf]

Supplementary Information

AK2 deficiency compromises the mitochondrial energy metabolism required for differentiation of human neutrophil and lymphoid lineages

Emmanuelle Six, Chantal Lagresle-Peyrou, Sandrine Susini, Corinne De Chappedelaine, Nelly Sigrist, Hanem Sadek, Myriam Chouteau, Nicolas Cagnard, Michaela Fontenay, O Hermine, Christine Chomienne, Pascal Reynier, Alain Fischer, Isabelle André-Schmutz, Naig Gueguen, Marina Cavazzana.

Supplementary Methods
Supplementary Figure S1
Supplementary Figure S2
Supplementary Figure S3
Supplementary Figure S4
Supplementary Figure S5
Supplementary Figure S6
Supplementary Figure S7
Supplementary Figure S8
Supplementary Table S1
Supplementary Table S2

Supplementary Methods

Constructs and vectors

We used the following target sequences for the shRNA: shAK2 #1 (ATGGTAGTGGAGCTCATTGAG), shAK2 #2 (GAAACTGGTGAGTGATGAAAT), shCont (CAACAAGATGAAGAGCACCAA). We generated pLKO-mCherry variants of the shAK2 #2 and shCont vectors by replacing the puromycin resistance cassette (BamHI and KpnI sites) by the mCherry reporter. hAK1 or hBcl2 cDNA were cloned into the pWPI lentiviral vector as previously described [1].

Double transduction of human CD34+ progenitor cells and the HL60 cell line

Cord blood CD34+ cells were cultured overnight (as previously described[1]) and transduced first with the pWPI supernatant and then twice with the pLKO-mCherry supernatant. Two days later, the GFP+mCherry+ cells were sorted on a FACSAria (BD Biosciences) prior to *in vitro* differentiation.

The HL60 cell line was transduced twice with pWPI supernatant in the presence of polybrene (4 µg/ml, Sigma Aldrich). In order to select cells expressing the lentiviral vector, GFP+ cells were sorted on a FACSAria (BD Biosciences). The HL60-GFP cell line was then transduced with the pLKO.1 lentiviral vector.

mRNA quantification

Total RNA was isolated with the RNeasy Micro Kit (Qiagen) and then reverse-transcribed using a High-capacity cDNA Reverse Transcription Kit (Life Technologies). Diluted cDNA was used as a template for quantitative, real-time polymerase chain reaction (PCR) reactions in TaqMan gene expression assays for AK2, AK1 and Bcl-2 (Hs01123132, Hs00176119 and Hs 00608023 respectively, Life Technologies). The transcript levels were normalized against the human glyceraldehyde-3-phosphate dehydrogenase (*GAPDH*, Hs02758991*)*. The PCRs were performed on a ViiA7 Real-Time PCR System (Applied Biosystems), according to the manufacturer's instructions.

Electron microscopy

Cells were fixed in medium and cultured for 15 min at 37◦C by the addition of an equal volume of 2x fixative solution [5% glutaraldehyde in PHEM buffer (60 mM Pipes, 25 mM Hepes, 10 mM EGTA and 2 mM MgCl2, pH=6.9)] and medium. Then cells were collected by

centrifugation for 5 min at 2000 g and fixation was continued for 1 h at room temperature with fresh fixative [2,5% (w/v) glutaraldehyde in PHEM buffer]. Then the cells were washed with PHEM buffer, and resuspended in 2,5% (w/v) glutaraldehyde in PHEM buffer and stored at 4◦C overnight. Cells were washed three times with PHEM buffer and were postfixed with 1% osmium tetroxide for 1 h at room temperature, dehydrated in ethanol gradient, and embedded in Epon. Thin sections were cut with a Leica EM UC7 Microtome, stained with uranyl acetate and lead citrate. The samples were observed under Tecnai T12 (FEI company) at 120 kV accelerating voltage. Images were recorded using Eagle camera and Tia software.

Bibliography

1 Lagresle-Peyrou C, Six EM, Picard C et al. Human adenylate kinase 2 deficiency causes a profound hematopoietic defect associated with sensorineural deafness. Nat Genet 2009;41:106-111.

Supplementary Figure 1

Six et al

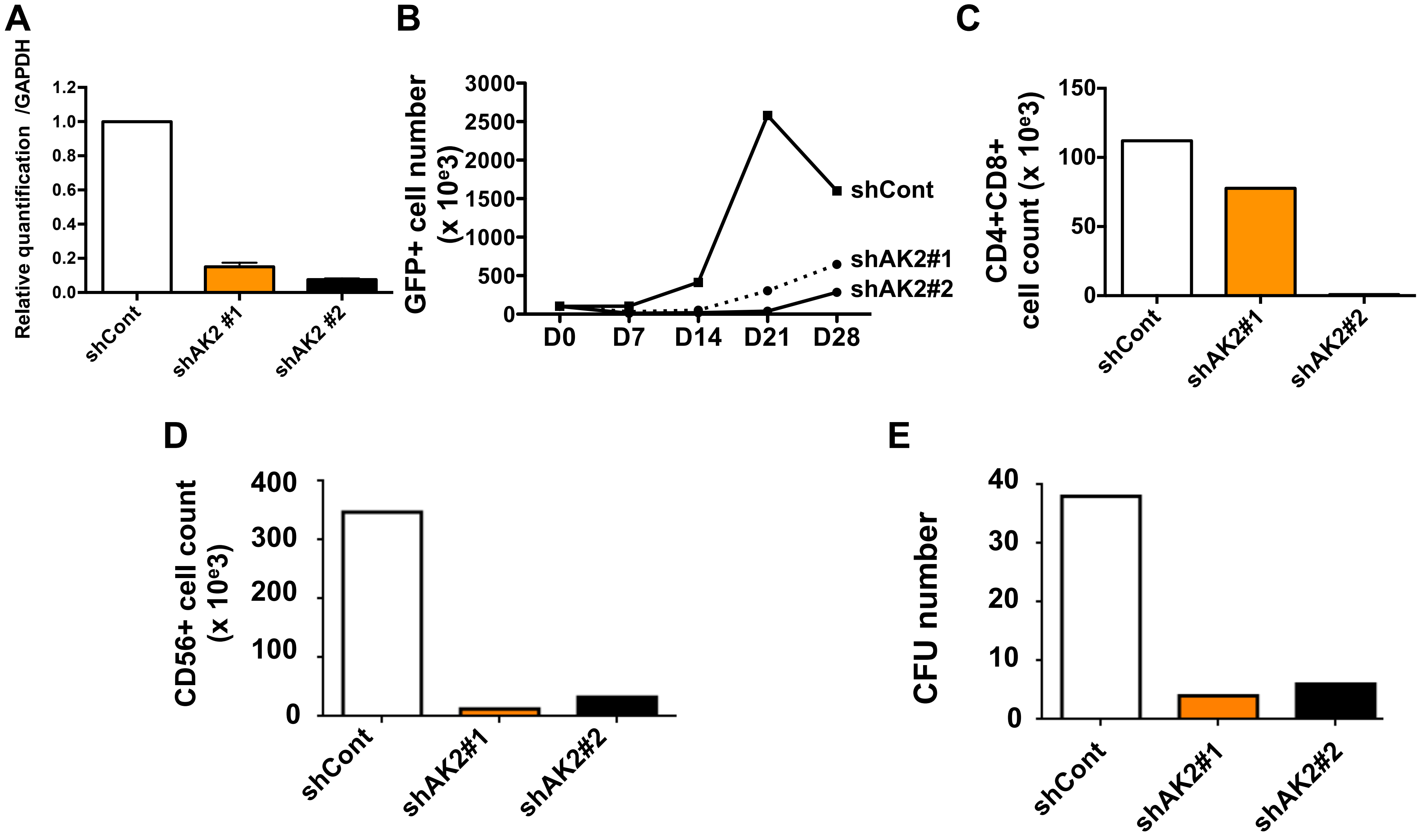

Figure S1

Two independent shAK2 approaches reveals similar degrees of inhibition of lymphoid and myeloid differentiation

Cord blood CD34+ progenitors were transduced with shAK2 #1, shAK2 #2 or shCont, sorted for GFP+ cells and cultured in transduction medium for 1 week (A) on OP9-hDelta1 cells until D28 (B-C), in NK cell differentiation medium until D21 (D) or seeded on methylcellulose until D12 (E).

(A) RT-qPCRs were performed on cell samples, in order to measure the level of AK2 inhibition.

(B) The change over time in the GFP+ cell count for shCont-transduced cells (black squares) or shAK2-transduced cells (shAK2 #1: dashed line black circles; shAK2#2: solid line black circles).

(C) The DP CD4+CD8+ cell count was determined under shCont, shAK2 #1 or shAK2#2 conditions after differentiation on OP9-hDelta1

(D) The CD56+ cell count was determined under shCont, shAK2 #1 or shAK2#2 condition after NK cell differentiation.

(E) The number of colony-forming units (CFUs) was evaluated in a methylcellulose assay under shCont, shAK2 #1 and shAK2#2 conditions. The CFUs include both CFU-G and CFU-M colonies.

Supplementary Figure 2

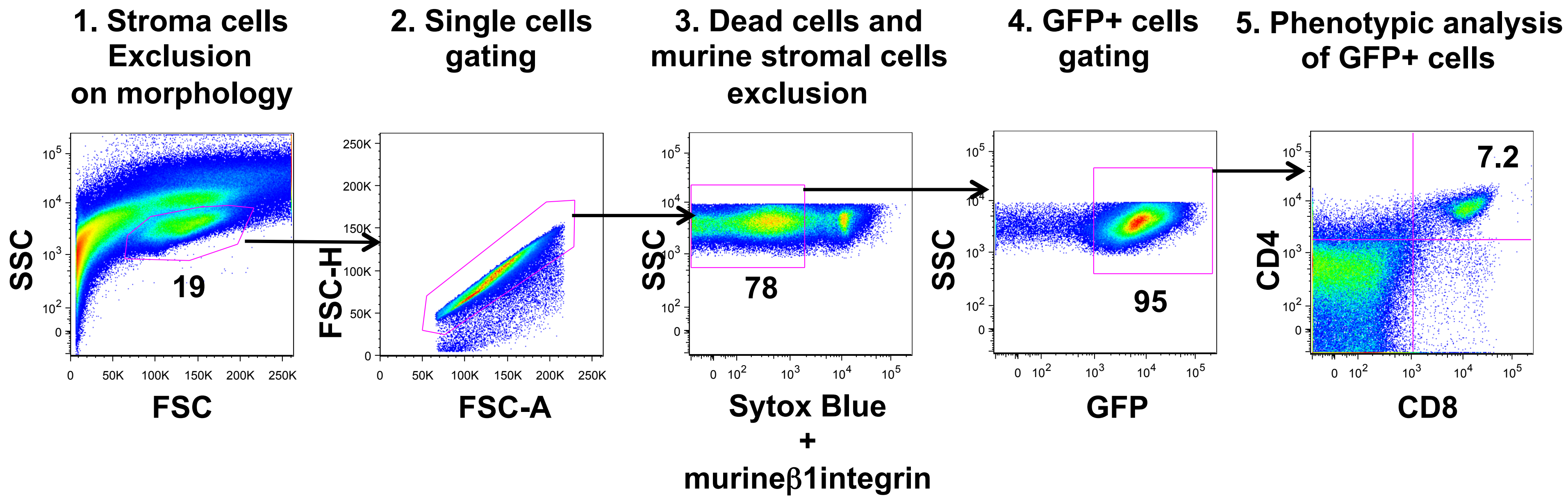

Figure S2

Flow cytometry gating strategy for T cell differentiation analysis of the GFP+ transduced cells.

Cord blood CD34+ progenitors were transduced with shCont, sorted for GFP+ cells and cultured for 28 days on OP9-hDelta1 stromal cell (to monitor T-cell differentiation).

In order to guarantee the removal of stromal cells (which are also GFP+), the flow cytometry analysis of the T-cell differentiation used a gating strategy with an antibody against murine b1 integrin. Dead cells were removed using Sytox Blue staining.

Six et al

Supplementary Figure 3

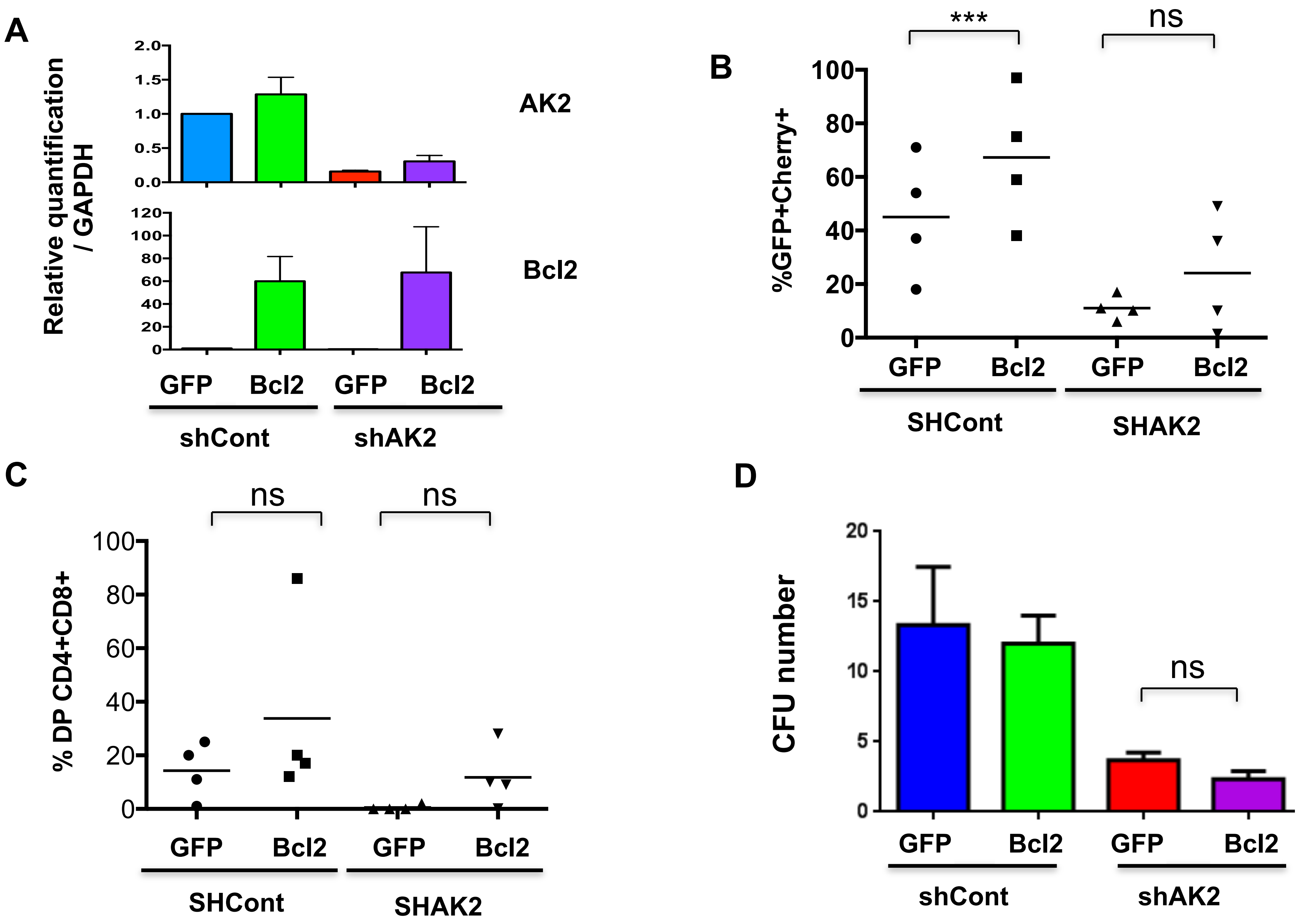

Figure S3
Bcl2 does not rescue survival and differentiation of AK2 knock-down progenitors.

Cord blood CD34+ progenitors were double-transduced with Bcl2 and shAK2 or shCont, sorted for GFP+mCherry+ cells and cultured in the transduction medium for 1 week (A), plated on OP9-hDelta1cells until D42 (B and C) or plated on methylcellulose for 12 days (D)

(A) RT-qPCRs were performed on cell samples, in order to measure the level of AK2 inhibition (upper panel, the slight increase in AK2 upon adition of Bcl2 is not significant) and monitor the expression of Bcl2 (lower panel)

(B) Percentage of GFP+mCherry+ cells 42 days after initiation of T cell differentiation under the shAK2 and shCont conditions (p=0.0002 between shCont+GFP and shCont + Bcl2).

(C) Percentage of DP CD4+CD8+ T cells 42 days after the initiation of T cell differentiation.

(D) The number of CFUs was evaluated in the shCont+GFP, shCont+Bcl2, shAK2+GFP and shAK2+Bcl2 conditions. The CFUs include both CFU-G and CFU-M colonies.

ns : not significant differences

Supplementary Figure 4

Six et al

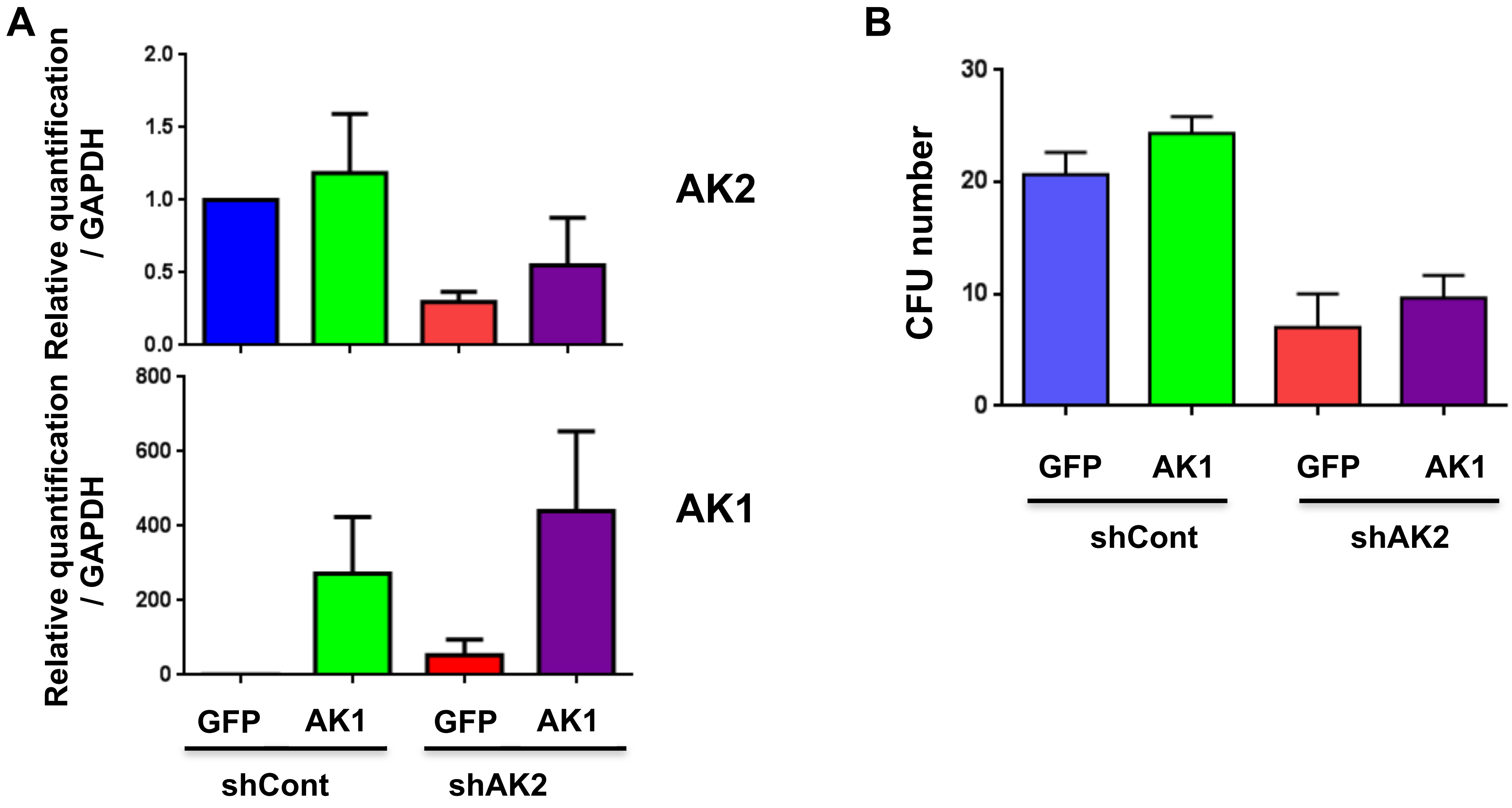

Figure S4

AK1 does not rescue survival and differentiation of AK2 knock-down progenitors

Cord blood CD34+ progenitors were double-transduced with AK1 and shAK2 or shCont, sorted for GFP+mCherry+ cells and cultured in the transduction medium for 1 week (A), or cultured on methylcellulose for 12 days (n=3) (B).

(A) RT-qPCR were performed on cell samples, in order to measure the level of AK2 inhibition (upper panel) and monitor the expression of AK1 (lower panel) under the shCont+GFP, shCont+AK1, shAK2+GFP and shAK2+AK1 conditions.

(B) The number of CFUs was evaluated under the shCont+GFP, shCont+AK1, shAK2+GFP and shAK2+AK1 conditions. The CFUs include both CFU-G and CFU-M colonies.

Supplementary Figure 5

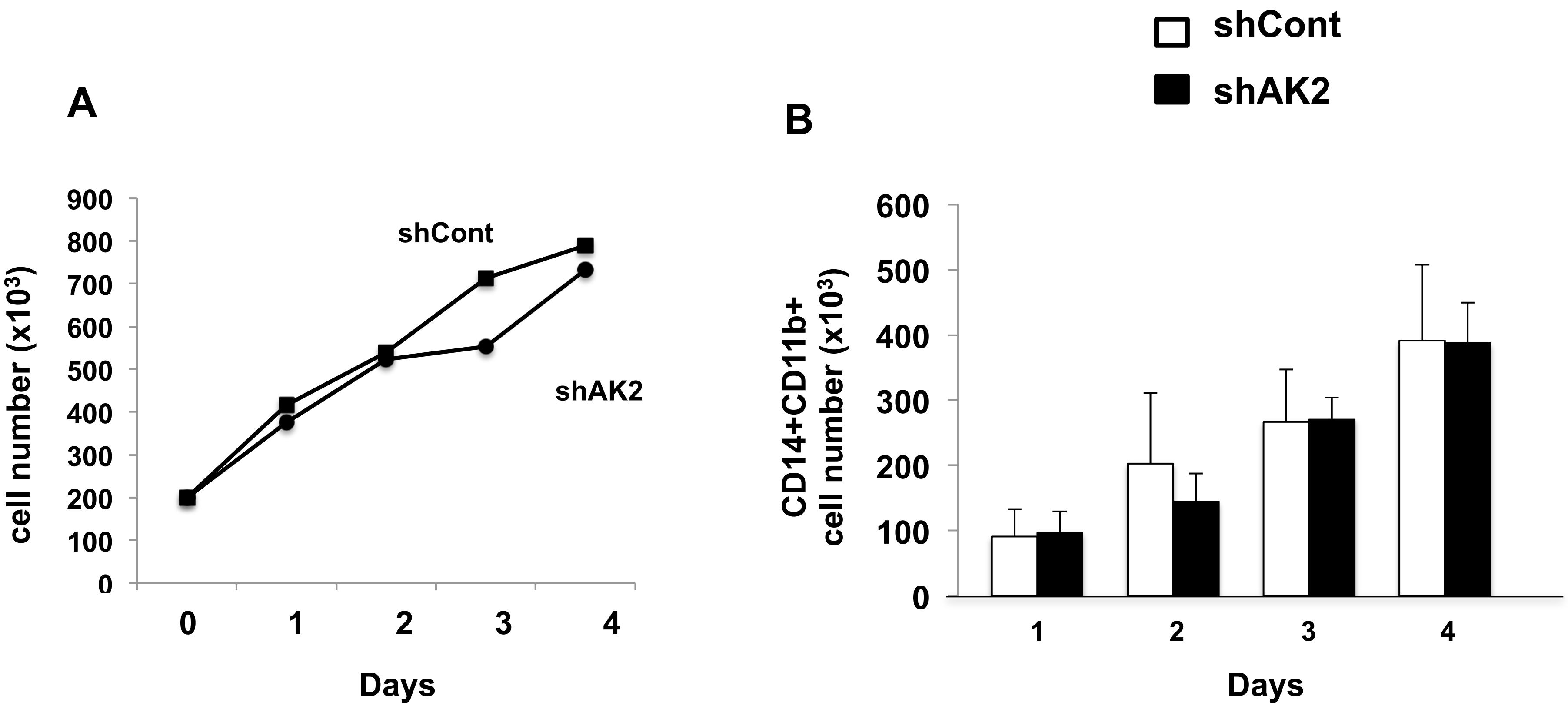

Figure S5
Monocyte differentiation is not impaired in the AK2-deficient HL60 cell line.
HL60 cells were transduced with shAK2 or shCont, sorted for GFP+ cells and seeded in culture with 100nM vitamin D3 (to induce monocyte differentiation).
(A) The change over time in the total cell count over 4 days of monocyte differentiation (n=4) under the shAK2 (black round) and shCont (black square) conditions.
(B) The CD14+CD11b+ monocyte count was measured for 4 days under the shAK2 (black bar) and shCont (white bar) conditions

Supplementary Figure S6

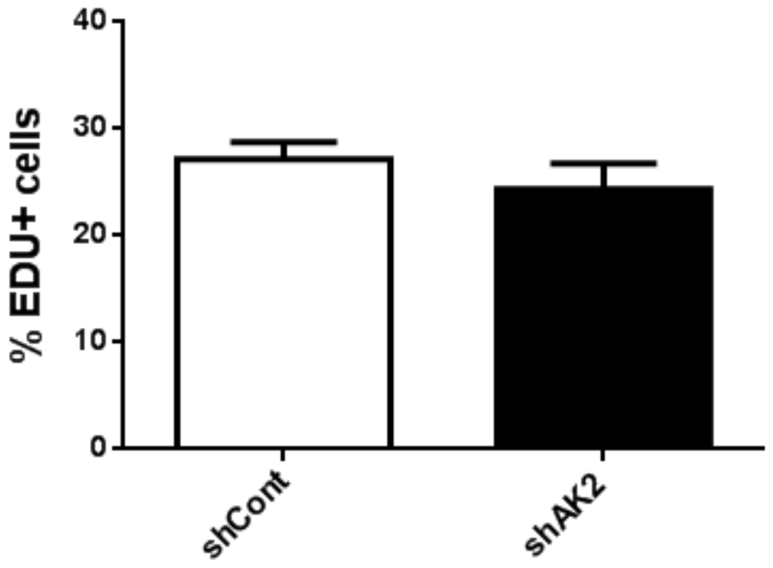

Figure S6

The proliferation of CD34 progenitors is not affected by AK2 knock-down

Cord blood CD34+ progenitors were transduced with shAK2 or shCont, sorted for GFP+ cells and cultured in the presence of SCF+TPO+FLT3-L+IL-3 (a cytokine cocktail commonly used to induce CD34+ cell proliferation). Cell proliferation (incorporation of EdU) was analyzed four days after the initiation of the culture under the shCont and shAK2 conditions ($p>0.05$, $n=3$).

Supplementary Figure 7

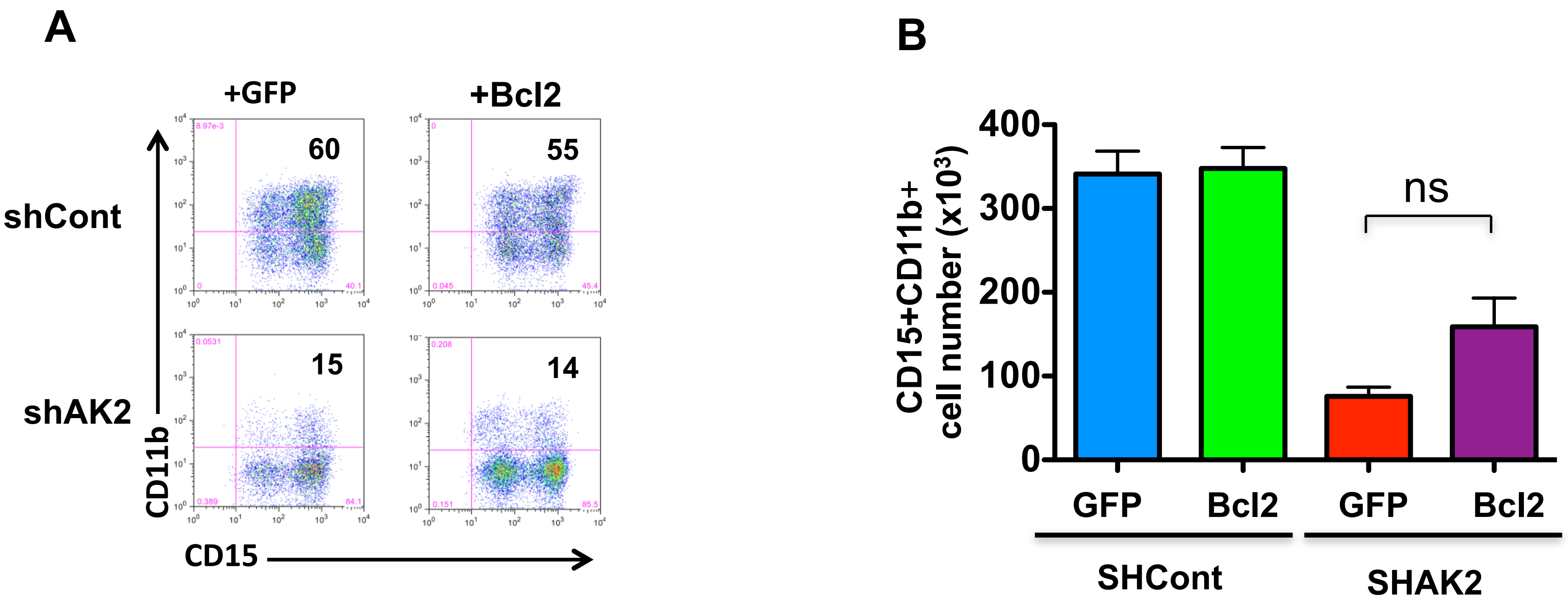

Figure S7

Bcl2 does not restore neutrophil differentiation of AK2-deficient HL60 cells.

HL60 cells were transduced with Bcl2 and shAK2 or shCont, selected for GFP+ and puromycin resistance and cultured with 10uM ATRA (to induce granulocyte differentiation).

(A) Flow cytometry analysis of GFP+ cells after 4 days of culture under shAK2 and shCont conditions.

(B) The CD15+CD11b+ granulocyte count was measured after 4 days under the shCont+GFP, shCont+Bcl2, shAK2+GFP and shAK2+Bcl2 conditions.

Supplementary Figure 8

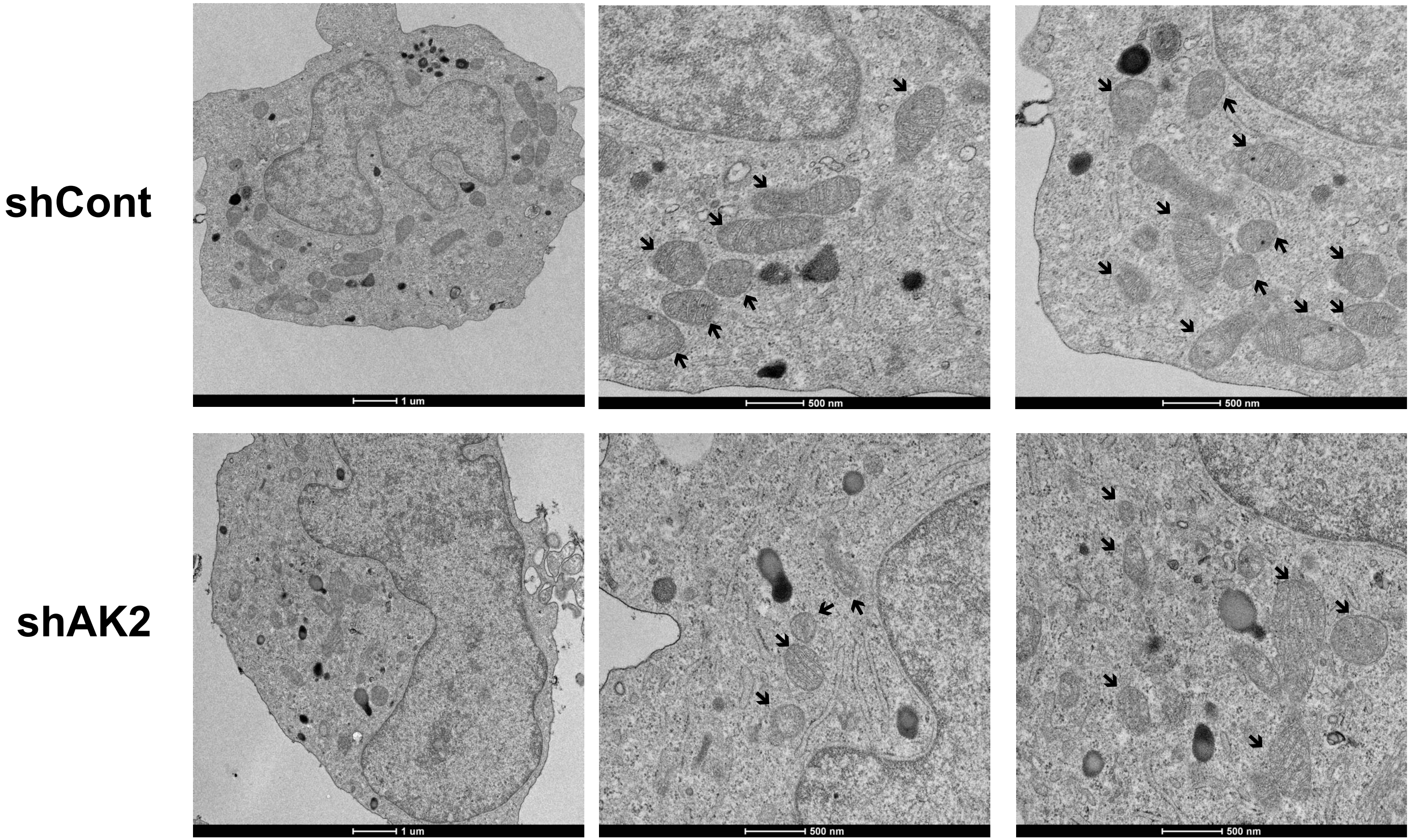

Figure S8
Mitochondria ultrastructure in AK2-deficient HL60 cells.
HL60 cells were transduced with shAK2 or shCont, selected for puromycin resistance and cultured with 10uM ATRA for 4 days (to induce granulocyte differentiation). Transmission electron microscopy revealed the mitochondrial ultrastructure. Arrow highlight mitochondria.

Supplementary Table S1
(the fold change (FC) and pvalue between shAK2 and shCont is shown for shAK2 #1 and shAK2 #2, upregulated genes are shown in bold type)

Gene Symbol	Entrez Gene Name	FC #1	FC #2	pvalue #1	pvalue #2
**AACS**	acetoacetyl-CoA synthetase	0.72	0.66	0.016	0.026
**ABCF2**	ATP-binding cassette, sub-family F (GCN20), member 2	0.62	0.78	0.035	0.032
**ABHD14A /// ACY1**	abhydrolase domain containing 14A /// aminoacylase 1	0.57	0.75	0.019	0.011
**ABHD14B**	abhydrolase domain containing 14B	0.81	0.61	0.037	0.033
**ACAD9**	acyl-CoA dehydrogenase family, member 9	0.80	0.71	0.027	0.011
**ACP5**	acid phosphatase 5, tartrate resistant	**4.43**	**7.22**	0.003	0.000
**ACSL3**	acyl-CoA synthetase long-chain family member 3	0.39	0.58	0.003	0.041
**ACSL5**	acyl-CoA synthetase long-chain family member 5	0.53	0.72	0.030	0.028
**ACSM3**	acyl-CoA synthetase medium-chain family member 3	**1.45**	**5.12**	0.042	0.003
**ACSS1**	acyl-CoA synthetase short-chain family member 1	0.58	0.62	0.040	0.043
**ACY3**	aspartoacylase (aminocyclase) 3	**1.30**	**3.54**	0.013	0.041
**ADCK2**	aarF domain containing kinase 2	0.65	0.53	0.022	0.011
**ADD2**	adducin 2 (beta)	0.18	0.13	0.009	0.004
**ADRBK2**	adrenergic, beta, receptor kinase 2	0.62	0.74	0.033	0.029
**AFF4**	AF4/FMR2 family, member 4	**1.30**	**2.16**	0.038	0.026
**AFG3L2**	AFG3 ATPase family gene 3-like 2 (S, cerevisiae)	0.73	0.76	0.008	0.020
**AHCYL1**	adenosylhomocysteinase-like 1	0.69	0.76	0.027	0.023
**AHSP**	alpha hemoglobin stabilizing protein	0.31	0.27	0.012	0.013
**AK2**	adenylate kinase 2	0.13	0.12	0.001	0.000
**AK7**	adenylate kinase 7	0.40	0.50	0.003	0.030
**AKAP13**	A kinase (PRKA) anchor protein 13	**1.28**	**1.35**	0.022	0.026
**AKIRIN1**	akirin 1	**1.56**	**1.54**	0.018	0.008
**AKR1C1**	aldo-keto reductase family 1, member C1	0.21	0.19	0.014	0.013
**AKR1C2**	aldo-keto reductase family 1, member C2	0.30	0.24	0.033	0.016
**AKT3**	v-akt murine thymoma viral oncogene homolog 3 (protein kinase B, gamma)	**1.71**	**1.92**	0.038	0.038
**ALAD**	aminolevulinate dehydratase	0.51	0.50	0.001	0.004
**ALAS2**	aminolevulinate, delta-, synthase 2	0.10	0.09	0.003	0.005
**ALOX5**	arachidonate 5-lipoxygenase	0.37	0.18	0.009	0.029
**AMMECR1**	Alport syndrome, mental retardation, midface hypoplasia and elliptocytosis chromosomal region gene 1	0.34	0.17	0.003	0.016
**ANK1**	ankyrin 1, erythrocytic	0.62	0.63	0.024	0.018
**ANKLE1**	ankyrin repeat and LEM domain containing 1	0.33	0.53	0.013	0.012
**ANKRD44**	ankyrin repeat domain 44	**1.84**	**1.57**	0.028	0.037
**ANPEP**	alanyl (membrane) aminopeptidase	**2.05**	**2.27**	0.004	0.041
**AP1M1**	adaptor-related protein complex 1, mu 1 subunit	0.80	0.82	0.032	0.023
**APOA1BP**	apolipoprotein A-I binding protein	0.58	0.80	0.001	0.007
**APOBEC3C**	apolipoprotein B mRNA editing enzyme, catalytic polypeptide-like 3C	0.68	0.83	0.025	0.005
**APOC1**	apolipoprotein C-I	0.21	0.31	0.005	0.027
**APOOL**	apolipoprotein O-like	0.70	0.69	0.012	0.030
**APP**	amyloid beta (A4) precursor protein	**1.66**	**1.49**	0.033	0.012
**APPL1**	adaptor protein, phosphotyrosine interaction, PH domain and leucine zipper containing 1	**1.52**	**1.86**	0.050	0.022
**ARHGAP19**	Rho GTPase activating protein 19	0.68	0.57	0.007	0.030
**ARHGEF3**	Rho guanine nucleotide exchange factor (GEF) 3	**2.42**	**3.05**	0.007	0.002
**ARHGEF37**	Rho guanine nucleotide exchange factor (GEF) 37	0.24	0.27	0.007	0.005
**ARID1A**	AT rich interactive domain 1A (SWI-like)	0.71	0.79	0.042	0.041
**ARPC3**	actin related protein 2/3 complex, subunit 3, 21kDa	**2.14**	**1.44**	0.030	0.043
**ARRDC2**	arrestin domain containing 2	**1.38**	**1.60**	0.025	0.047
**ARSB**	arylsulfatase B	0.56	0.53	0.042	0.037

**ASAH1**	N-acylsphingosine amidohydrolase (acid ceramidase) 1	**1.57**	**1.98**	0.034	0.008
**ASNA1**	arsA arsenite transporter, ATP-binding, homolog 1 (bacterial)	0.83	0.62	0.034	0.002
**ASPM**	asp (abnormal spindle) homolog, microcephaly associated (Drosophila)	0.76	0.61	0.025	0.007
**ASRGL1**	asparaginase like 1	0.57	0.54	0.035	0.024
**ATAD3A**	ATPase family, AAA domain containing 3A	0.80	0.80	0.017	0.023
**ATAD3B**	ATPase family, AAA domain containing 3B	0.77	0.76	0.045	0.031
**ATP8B4**	ATPase, class I, type 8B, member 4	**2.04**	**1.48**	0.038	0.036
**ATPIF1**	ATPase inhibitory factor 1	0.49	0.43	0.031	0.010
**AUH**	AU RNA binding protein/enoyl-CoA hydratase	**1.40**	**1.52**	0.006	0.014
**AURKA**	aurora kinase A	0.78	0.59	0.012	0.005
**AVL9**	AVL9 homolog (S, cerevisiase)	0.70	0.49	0.049	0.008
**B2M**	beta-2-microglobulin	**1.30**	**1.25**	0.023	0.050
**BAALC**	brain and acute leukemia, cytoplasmic	**2.45**	**4.27**	0.040	0.004
**BANF1**	barrier to autointegration factor 1	0.70	0.70	0.009	0.017
**BAZ2B**	bromodomain adjacent to zinc finger domain, 2B	**2.04**	**2.26**	0.031	0.019
**BCL2L11**	BCL2-like 11 (apoptosis facilitator)	0.61	0.43	0.014	0.018
**BCS1L**	BCS1-like (S, cerevisiae)	0.74	0.61	0.019	0.013
**BIRC2**	baculoviral IAP repeat-containing 2	**1.29**	**1.42**	0.042	0.027
**BLVRB**	biliverdin reductase B (flavin reductase (NADPH))	0.26	0.18	0.002	0.011
**BOLA3**	bolA homolog 3 (E, coli)	0.26	0.69	0.001	0.002
**BOP1**	block of proliferation 1	0.66	0.65	0.030	0.026
**BRP44**	brain protein 44	0.53	0.42	0.000	0.032
**BTBD11**	BTB (POZ) domain containing 11	**1.71**	**1.87**	0.008	0.019
**BTBD6**	BTB (POZ) domain containing 6	0.70	0.62	0.018	0.038
**BTG1**	B-cell translocation gene 1, anti-proliferative	**1.57**	**1.55**	0.002	0.026
**C10orf46**	chromosome 10 open reading frame 46	**1.58**	**1.46**	0.000	0.030
**C12orf43**	chromosome 12 open reading frame 43	0.69	0.73	0.024	0.041
**C12orf52**	chromosome 12 open reading frame 52	0.61	0.79	0.011	0.008
**C14orf135**	chromosome 14 open reading frame 135	0.55	0.55	0.022	0.028
**C16orf88**	chromosome 16 open reading frame 88	0.62	0.69	0.022	0.007
**C17orf90**	chromosome 17 open reading frame 90	0.79	0.71	0.032	0.006
**C17orf99**	chromosome 17 open reading frame 99	0.18	0.14	0.006	0.005
**C18orf10**	chromosome 18 open reading frame 10	0.50	0.57	0.011	0.026
**C1orf135**	chromosome 1 open reading frame 135	0.83	0.79	0.027	0.006
**C1orf162**	chromosome 1 open reading frame 162	0.52	0.65	0.033	0.023
**C1orf198**	chromosome 1 open reading frame 198	0.51	0.50	0.002	0.007
**C1orf93**	chromosome 1 open reading frame 93	0.57	0.62	0.038	0.039
**C1QBP**	complement component 1, q subcomponent binding protein	0.76	0.77	0.002	0.004
**C20orf108**	chromosome 20 open reading frame 108	0.40	0.38	0.009	0.005
**C20orf194**	chromosome 20 open reading frame 194	**2.02**	**2.66**	0.017	0.027
**C21orf91**	chromosome 21 open reading frame 91	**1.60**	**1.94**	0.023	0.010
**C22orf13**	chromosome 22 open reading frame 13	0.66	0.66	0.002	0.009
**C3orf17**	chromosome 3 open reading frame 17	**1.23**	**1.30**	0.010	0.000
**C3orf39**	chromosome 3 open reading frame 39	0.50	0.54	0.009	0.047
**C5orf33**	chromosome 5 open reading frame 33	0.69	0.53	0.039	0.039
**C5orf62**	chromosome 5 open reading frame 62	**2.35**	**1.69**	0.012	0.027
**C6orf108**	chromosome 6 open reading frame 108	0.52	0.61	0.032	0.013
**C6orf89**	chromosome 6 open reading frame 89	0.47	0.46	0.018	0.034
**C7orf44**	chromosome 7 open reading frame 44	0.43	0.66	0.004	0.025
**C8orf30A**	chromosome 8 open reading frame 30A	0.75	0.74	0.016	0.039
**C8orf82**	chromosome 8 open reading frame 82	0.68	0.62	0.027	0.022
**C9orf86**	chromosome 9 open reading frame 86	0.58	0.43	0.008	0.007
**CACNA2D4**	calcium channel, voltage-dependent, alpha 2/delta subunit 4	0.61	0.62	0.023	0.043
**CALML4**	calmodulin-like 4	**1.92**	**1.71**	0.019	0.023
**CAND1**	cullin-associated and neddylation-dissociated 1	0.60	0.46	0.041	0.020
**CARKD**	carbohydrate kinase domain containing	0.69	0.57	0.022	0.001
**CARS2**	cysteinyl-tRNA synthetase 2, mitochondrial (putative)	0.80	0.65	0.017	0.005

**CAST**	calpastatin	0.37	0.45	0.005	0.014
**CBARA1**	calcium binding atopy-related autoantigen 1	0.70	0.68	0.040	0.037
**CBX5**	chromobox homolog 5	0.68	0.77	0.028	0.049
**CCDC77**	coiled-coil domain containing 77	0.81	0.51	0.025	0.020
**CCDC90B**	coiled-coil domain containing 90B	**1.64**	**2.51**	0.010	0.006
**CCNA1**	cyclin A1	**4.09**	**1.70**	0.001	0.026
**CCNE1**	cyclin E1	0.64	0.41	0.001	0.003
**CCNF**	cyclin F	0.63	0.49	0.020	0.035
**CCR2**	chemokine (C-C motif) receptor 2	0.56	0.49	0.009	0.049
**CCT5**	chaperonin containing TCP1, subunit 5 (epsilon)	0.43	0.50	0.007	0.007
**CCT7**	chaperonin containing TCP1, subunit 7 (eta)	0.82	0.83	0.008	0.014
**CD109**	CD109 molecule	**2.08**	**7.22**	0.005	0.014
**CD1D**	CD1d molecule	**1.79**	**2.83**	0.044	0.015
**CD200**	CD200 molecule	**2.91**	**9.96**	0.007	0.028
**CD300A**	CD300a molecule	**1.21**	**1.43**	0.048	0.019
**CD302**	CD302 molecule	**1.47**	**1.36**	0.007	0.029
**CD34**	CD34 molecule	**1.88**	**2.67**	0.001	0.036
**CD44**	CD44 molecule (Indian blood group)	**1.43**	**1.88**	0.038	0.037
**CD48**	CD48 molecule	**1.48**	**1.79**	0.019	0.025
**CDC20**	cell division cycle 20 homolog (S, cerevisiae)	0.62	0.46	0.043	0.002
**CDC27**	cell division cycle 27 homolog (S, cerevisiae)	0.78	0.37	0.003	0.007
**CDC45**	cell division cycle 45 homolog (S, cerevisiae)	0.67	0.50	0.030	0.017
**CDC6**	cell division cycle 6 homolog (S, cerevisiae)	0.59	0.55	0.020	0.019
**CDH1**	cadherin 1, type 1, E-cadherin (epithelial)	0.47	0.09	0.027	0.002
**CDK10**	cyclin-dependent kinase 10	0.57	0.72	0.003	0.038
**CDK5RAP2**	CDK5 regulatory subunit associated protein 2	0.59	0.52	0.009	0.032
**CDKN1C**	cyclin-dependent kinase inhibitor 1C (p57, Kip2)	**4.17**	**8.12**	0.001	0.002
**CEBPB**	CCAAT/enhancer binding protein (C/EBP), beta	**1.42**	**1.87**	0.022	0.010
**CENPN**	centromere protein N	0.82	0.73	0.041	0.041
**CENPV**	centromere protein V	0.42	0.60	0.004	0.045
**CEP72**	centrosomal protein 72kDa	0.50	0.60	0.029	0.045
**CFH**	complement factor H	**4.52**	**3.23**	0.008	0.005
**CFH /// CFHR1**	complement factor H /// complement factor H-related 1	**2.75**	**10.89**	0.002	0.001
**CHCHD4**	coiled-coil-helix-coiled-coil-helix domain containing 4	0.78	0.67	0.023	0.014
**CHD7**	chromodomain helicase DNA binding protein 7	0.58	0.74	0.028	0.039
**CHEK2**	CHK2 checkpoint homolog (S, pombe)	0.71	0.66	0.018	0.015
**CHPT1**	choline phosphotransferase 1	0.75	0.54	0.048	0.011
**CKAP2L**	cytoskeleton associated protein 2-like	0.73	0.58	0.046	0.046
**CLC**	Charcot-Leyden crystal protein	0.25	0.06	0.009	0.017
**CLDN10**	claudin 10	**2.71**	**3.41**	0.012	0.006
**CLEC2B**	C-type lectin domain family 2, member B	**3.70**	**3.53**	0.006	0.020
**CLIC4**	chloride intracellular channel 4	**1.71**	**1.54**	0.007	0.001
**CLN6**	ceroid-lipofuscinosis, neuronal 6, late infantile, variant	0.59	0.72	0.006	0.027
**CLU**	clusterin	0.48	0.66	0.017	0.018
**CMAH**	cytidine monophosphate-N-acetylneuraminic acid hydroxylase (CMP-N-acetylneuraminate monooxygenase) pseudogene	**4.04**	**3.34**	0.043	0.042
**CNST**	consortin, connexin sorting protein	**1.73**	**2.72**	0.031	0.026
**COASY**	CoA synthase	0.71	0.75	0.050	0.003
**COCH**	coagulation factor C homolog, cochlin (Limulus polyphemus)	0.27	0.31	0.013	0.023
**COPS6**	COP9 constitutive photomorphogenic homolog subunit 6 (Arabidopsis)	0.56	0.68	0.010	0.030
**COQ3**	coenzyme Q3 homolog, methyltransferase (S, cerevisiae)	0.60	0.66	0.023	0.023
**COTL1**	coactosin-like 1 (Dictyostelium)	0.63	0.59	0.008	0.008
**COX5A**	cytochrome c oxidase subunit Va	0.55	0.57	0.012	0.047
**CPNE8**	copine VIII	**1.56**	**2.52**	0.013	0.018
**CRHBP**	corticotropin releasing hormone binding protein	**3.63**	**19.17**	0.011	0.019
**CRIM1**	cysteine rich transmembrane BMP regulator 1 (chordin-like)	**1.80**	**3.21**	0.026	0.024

**CRYZL1**	crystallin, zeta (quinone reductase)-like 1	**1.38**	**1.38**	0.028	0.007
**CSRP1**	cysteine and glycine-rich protein 1	0.75	0.77	0.007	0.037
**CTHRC1**	collagen triple helix repeat containing 1	**3.88**	**2.96**	0.012	0.038
**CTNNAL1**	catenin (cadherin-associated protein), alpha-like 1	0.72	0.43	0.041	0.018
**CTNNB1**	catenin (cadherin-associated protein), beta 1, 88kDa	**2.46**	**2.13**	0.014	0.016
**CTSC**	cathepsin C	0.71	0.55	0.025	0.019
**CTSE**	cathepsin E	0.30	0.27	0.032	0.048
**CTTN**	cortactin	0.16	0.25	0.005	0.025
**CUL4B**	cullin 4B	**1.75**	**1.33**	0.026	0.006
**CXADR**	coxsackie virus and adenovirus receptor	0.21	0.30	0.003	0.026
**CYP1B1**	cytochrome P450, family 1, subfamily B, polypeptide 1	**2.06**	**4.68**	0.044	0.029
**CYSLTR1**	cysteinyl leukotriene receptor 1	**1.94**	**2.34**	0.042	0.031
**CYYR1**	cysteine/tyrosine-rich 1	**2.13**	**4.87**	0.044	0.009
**DAAM1**	dishevelled associated activator of morphogenesis 1	0.38	0.36	0.022	0.014
**DAZAP1**	DAZ associated protein 1	0.77	0.70	0.039	0.028
**DCBLD2**	Discoidin, CUB and LCCL domain containing 2	**4.28**	**4.04**	0.008	0.027
**DCLRE1B**	DNA cross-link repair 1B	0.77	0.63	0.027	0.006
**DCUN1D5**	DCN1, defective in cullin neddylation 1, domain containing 5 (S, cerevisiae)	0.80	0.59	0.049	0.031
**DDAH2**	dimethylarginine dimethylaminohydrolase 2	**1.75**	**2.18**	0.008	0.014
**DDI2**	DNA-damage inducible 1 homolog 2 (S, cerevisiae)	0.46	0.45	0.009	0.025
**DDX11 /// DDX12 /// LOC642846**	DEAD/H (Asp-Glu-Ala-Asp/His) box polypeptide 11 /// DEAD/H (Asp-Glu-Ala-Asp/His) box polypeptide 12 /// DEAD/H (Asp-Glu-Ala-Asp/His) box polypeptide 11-like	0.76	0.39	0.010	0.002
**DDX24**	DEAD (Asp-Glu-Ala-Asp) box polypeptide 24	0.74	0.79	0.010	0.003
**DDX39**	DEAD (Asp-Glu-Ala-Asp) box polypeptide 39	0.69	0.59	0.013	0.006
**DDX41**	DEAD (Asp-Glu-Ala-Asp) box polypeptide 41	0.78	0.70	0.025	0.007
**DHCR7**	7-dehydrocholesterol reductase	0.60	0.51	0.009	0.004
**DHFR**	dihydrofolate reductase	0.62	0.73	0.001	0.034
**DHRS11**	dehydrogenase/reductase (SDR family) member 11	0.14	0.22	0.002	0.006
**DHX35**	DEAH (Asp-Glu-Ala-His) box polypeptide 35	0.68	0.69	0.028	0.039
**DLD**	dihydrolipoamide dehydrogenase	0.67	0.60	0.034	0.017
**DLST**	dihydrolipoamide S-succinyltransferase (E2 component of 2-oxo-glutarate complex)	0.68	0.80	0.001	0.005
**DMXL2**	Dmx-like 2	**3.32**	**1.69**	0.009	0.015
**DNAJA4**	DnaJ (Hsp40) homolog, subfamily A, member 4	0.35	0.30	0.017	0.014
**DNAJC10**	DnaJ (Hsp40) homolog, subfamily C, member 10	**1.75**	**1.78**	0.035	0.019
**DNAJC11**	DnaJ (Hsp40) homolog, subfamily C, member 11	0.71	0.76	0.007	0.009
**DNAJC19**	DnaJ (Hsp40) homolog, subfamily C, member 19	0.66	0.78	0.018	0.018
**DNAJC9**	DnaJ (Hsp40) homolog, subfamily C, member 9	0.80	0.74	0.025	0.044
**DNASE2**	deoxyribonuclease II, lysosomal	**1.40**	**2.66**	0.030	0.045
**DOK4**	docking protein 4	**2.58**	**4.43**	0.014	0.011
**DOLPP1**	dolichyl pyrophosphate phosphatase 1	0.72	0.77	0.027	0.026
**DONSON**	downstream neighbor of SON	0.68	0.50	0.027	0.001
**DPH5**	DPH5 homolog (S, cerevisiae)	**1.61**	**1.87**	0.015	0.010
**DPP3**	dipeptidyl-peptidase 3	0.66	0.77	0.027	0.048
**DPP7**	Dipeptidyl-peptidase 7	0.23	0.23	0.000	0.005
**DPYSL2**	dihydropyrimidinase-like 2	0.70	0.50	0.030	0.019
**DRG1**	developmentally regulated GTP binding protein 1	0.82	0.82	0.017	0.007
**DSC2**	desmocollin 2	**4.35**	**2.51**	0.029	0.045
**DTYMK**	deoxythymidylate kinase (thymidylate kinase)	0.61	0.57	0.023	0.036
**EARS2**	glutamyl-tRNA synthetase 2, mitochondrial (putative)	0.66	0.67	0.046	0.046
**EEFSEC**	eukaryotic elongation factor, selenocysteine-tRNA-specific	0.59	0.71	0.007	0.018
**EHD4**	EH-domain containing 4	0.70	0.53	0.036	0.002
**EIF2AK1**	eukaryotic translation initiation factor 2-alpha kinase 1	0.58	0.70	0.001	0.002
**EIF3B**	eukaryotic translation initiation factor 3, subunit B	0.73	0.71	0.024	0.029
**EIF6**	eukaryotic translation initiation factor 6	0.57	0.80	0.003	0.022
**ELOVL1**	elongation of very long chain fatty acids (FEN1/Elo2, SUR4/Elo3, yeast)-like 1	0.73	0.70	0.040	0.038

**EME1**	essential meiotic endonuclease 1 homolog 1 (S, pombe)	0.60	0.62	0.005	0.009
**EMP2**	epithelial membrane protein 2	0.31	0.35	0.006	0.012
**ENDOD1**	endonuclease domain containing 1	0.43	0.61	0.002	0.039
**ENDOG**	endonuclease G	0.69	0.58	0.025	0.008
**ENO3**	enolase 3 (beta, muscle)	0.73	0.58	0.032	0.018
**ENOSF1**	enolase superfamily member 1	0.60	0.56	0.013	0.006
**EPB42**	erythrocyte membrane protein band 4,2	0.25	0.29	0.023	0.031
**EPC2**	enhancer of polycomb homolog 2 (Drosophila)	**1.35**	**1.58**	0.012	0.013
**EPCAM**	epithelial cell adhesion molecule	0.18	0.17	0.007	0.023
**EPM2AIP1**	EPM2A (laforin) interacting protein 1	0.79	0.71	0.008	0.013
**EPOR**	erythropoietin receptor	0.33	0.30	0.009	0.007
**EPS15L1**	epidermal growth factor receptor pathway substrate 15-like 1	0.64	0.47	0.031	0.019
**ERAL1**	Era G-protein-like 1 (E, coli)	0.57	0.79	0.013	0.049
**ERG**	v-ets erythroblastosis virus E26 oncogene homolog (avian)	**1.74**	**3.43**	0.020	0.018
**ERI1**	exoribonuclease 1	0.50	0.56	0.003	0.016
**ERMAP**	erythroblast membrane-associated protein (Scianna blood group)	0.47	0.62	0.001	0.045
**ETV6**	ets variant 6	**1.38**	**1.64**	0.036	0.023
**EXOSC2**	exosome component 2	0.72	0.78	0.038	0.026
**EXOSC4**	exosome component 4	0.68	0.82	0.025	0.049
**EZR**	ezrin	0.54	0.67	0.011	0.022
**F2RL1**	coagulation factor II (thrombin) receptor-like 1	**1.34**	**6.65**	0.037	0.011
**FADS1**	fatty acid desaturase 1	0.61	0.63	0.018	0.009
**FAF1**	Fas (TNFRSF6) associated factor 1	0.79	0.62	0.005	0.002
**FAM178B**	family with sequence similarity 178, member B	0.33	0.34	0.005	0.006
**FAM26F**	family with sequence similarity 26, member F	**1.31**	**2.06**	0.016	0.035
**FAM46C**	family with sequence similarity 46, member C	0.58	0.28	0.012	0.003
**FAM60A**	family with sequence similarity 60, member A	**1.34**	**1.53**	0.029	0.012
**FAM82A2**	family with sequence similarity 82, member A2	0.76	0.65	0.021	0.003
**FAM89A**	family with sequence similarity 89, member A	0.19	0.32	0.006	0.005
**FANCD2**	Fanconi anemia, complementation group D2	0.69	0.46	0.008	0.024
**FANCI**	Fanconi anemia, complementation group I	0.71	0.53	0.009	0.017
**FARSB**	phenylalanyl-tRNA synthetase, beta subunit	0.74	0.60	0.021	0.023
**FBXL4**	F-box and leucine-rich repeat protein 4	**1.58**	**1.43**	0.037	0.030
**FBXO4**	F-box protein 4	0.58	0.69	0.008	0.006
**FBXO7**	F-box protein 7	0.59	0.57	0.035	0.035
**FBXO9**	F-box protein 9	0.66	0.70	0.005	0.013
**FBXW2**	F-box and WD repeat domain containing 2	0.80	0.82	0.014	0.024
**FCGR1B**	Fc fragment of IgG, high affinity Ib, receptor (CD64)	**2.76**	**3.68**	0.007	0.000
**FDPS**	farnesyl diphosphate synthase	0.68	0.63	0.006	0.004
**FECH**	ferrochelatase	0.34	0.20	0.010	0.003
**FGFR3**	fibroblast growth factor receptor 3	0.28	0.38	0.003	0.012
**FIBP**	fibroblast growth factor (acidic) intracellular binding protein	0.58	0.81	0.015	0.008
**FKBP4**	FK506 binding protein 4, 59kDa	0.54	0.67	0.009	0.044
**FKBP5**	FK506 binding protein 5	0.78	0.77	0.048	0.019
**FKBPL**	FK506 binding protein like	0.61	0.64	0.000	0.035
**FLI1**	Friend leukemia virus integration 1	**1.76**	**2.49**	0.032	0.011
**FLOT2**	flotillin 2	0.36	0.69	0.011	0.024
**FN3KRP**	fructosamine 3 kinase related protein	0.64	0.65	0.001	0.015
**FOXM1**	forkhead box M1	0.36	0.49	0.013	0.001
**FTH1**	ferritin, heavy polypeptide 1	0.75	0.58	0.015	0.022
**FZD3**	frizzled homolog 3 (Drosophila)	0.44	0.43	0.021	0.022
**GALNT14**	UDP-N-acetyl-alpha-D-galactosamine:polypeptide N-acetylgalactosaminyltransferase 14 (GalNAc-T14)	**1.37**	**2.15**	0.006	0.031
**GARS**	glycyl-tRNA synthetase	0.68	0.70	0.005	0.001
**GAS2L1**	growth arrest-specific 2 like 1	0.42	0.42	0.037	0.032
**GATA1**	GATA binding protein 1 (globin transcription factor 1)	0.27	0.30	0.004	0.010
**GBAP1**	glucosidase, beta, acid pseudogene 1	0.55	0.70	0.014	0.029

**GBGT1**	globoside alpha-1,3-N-acetylgalactosaminyltransferase 1	0.30	0.55	0.008	0.005
**GBP2**	guanylate binding protein 2, interferon-inducible	**4.67**	**3.87**	0.002	0.002
**GBP3**	guanylate binding protein 3	**2.69**	**4.30**	0.005	0.005
**GFI1**	growth factor independent 1 transcription repressor	**1.66**	**2.46**	0.013	0.003
**GFI1B**	growth factor independent 1B transcription repressor	0.25	0.45	0.012	0.037
**GGA2**	golgi-associated, gamma adaptin ear containing, ARF binding protein 2	0.80	0.82	0.007	0.016
**GIMAP1**	GTPase, IMAP family member 1	**2.07**	**2.29**	0.043	0.002
**GIMAP4**	GTPase, IMAP family member 4	**2.84**	**4.24**	0.007	0.023
**GIMAP5**	GTPase, IMAP family member 5	**2.84**	**5.03**	0.008	0.000
**GIMAP6**	GTPase, IMAP family member 6	**1.88**	**4.39**	0.029	0.012
**GIMAP7**	GTPase, IMAP family member 7	**2.29**	**3.35**	0.009	0.000
**GIMAP8**	GTPase, IMAP family member 8	**2.67**	**5.99**	0.004	0.018
**GINS2**	GINS complex subunit 2 (Psf2 homolog)	0.61	0.53	0.005	0.014
**GLCCI1**	glucocorticoid induced transcript 1	0.46	0.49	0.029	0.024
**GLIPR1**	GLI pathogenesis-related 1	**1.53**	**1.86**	0.019	0.030
**GLRX**	glutaredoxin (thioltransferase)	0.71	0.78	0.023	0.025
**GLRX5**	glutaredoxin 5	0.68	0.73	0.029	0.029
**GLT25D2**	glycosyltransferase 25 domain containing 2	0.71	0.70	0.018	0.017
**GNAI1**	guanine nucleotide binding protein (G protein), alpha inhibiting activity polypeptide 1	**2.54**	**3.67**	0.036	0.016
**GNS**	glucosamine (N-acetyl)-6-sulfatase	**1.54**	**3.76**	0.010	0.019
**GOSR2**	golgi SNAP receptor complex member 2	0.73	0.77	0.003	0.018
**GOT2**	glutamic-oxaloacetic transaminase 2, mitochondrial (aspartate aminotransferase 2)	0.67	0.80	0.012	0.017
**GPD1L**	glycerol-3-phosphate dehydrogenase 1-like	0.66	0.78	0.021	0.026
**GPX1**	glutathione peroxidase 1	**1.23**	**1.34**	0.001	0.010
**GRK6**	G protein-coupled receptor kinase 6	0.79	0.76	0.043	0.033
**GRTP1**	growth hormone regulated TBC protein 1	0.15	0.12	0.003	0.001
**GSS**	glutathione synthetase	0.66	0.78	0.021	0.008
**GTDC1**	glycosyltransferase-like domain containing 1	**1.24**	**1.72**	0.031	0.031
**GTSE1**	G-2 and S-phase expressed 1	0.53	0.52	0.006	0.012
**GUCY1A3**	guanylate cyclase 1, soluble, alpha 3	**2.97**	**4.67**	0.006	0.016
**GYPA**	glycophorin A (MNS blood group)	0.15	0.16	0.017	0.019
**HAGH**	hydroxyacylglutathione hydrolase	0.52	0.44	0.039	0.000
**HBA1 /// HBA2**	hemoglobin, alpha 1 /// hemoglobin, alpha 2	0.07	0.02	0.005	0.001
**HBB**	hemoglobin, beta	0.19	0.04	0.014	0.027
**HBD**	hemoglobin, delta	0.18	0.05	0.015	0.016
**HBG1 /// HBG2**	hemoglobin, gamma A /// hemoglobin, gamma G	0.05	0.04	0.008	0.030
**HBP1**	HMG-box transcription factor 1	**1.46**	**1.84**	0.002	0.003
**HBS1L**	HBS1-like (S, cerevisiae)	0.59	0.55	0.017	0.013
**HDHD3**	haloacid dehalogenase-like hydrolase domain containing 3	0.54	0.60	0.028	0.005
**HEBP1**	heme binding protein 1	0.51	0.36	0.000	0.002
**HEBP2**	heme binding protein 2	**1.20**	**1.51**	0.043	0.026
**HERC6**	hect domain and RLD 6	**1.31**	**2.42**	0.033	0.036
**HIRIP3**	HIRA interacting protein 3	0.72	0.58	0.043	0.002
**HIST3H2A**	histone cluster 3, H2a	0.54	0.56	0.044	0.047
**HJURP**	Holliday junction recognition protein	0.80	0.60	0.019	0.020
**HK1**	hexokinase 1	0.59	0.73	0.001	0.027
**HLA-DQB1 /// LOC100133583**	major histocompatibility complex, class II, DQ beta 1 /// HLA class II histocompatibility antigen, DQ beta 1 chain-like	0.61	0.22	0.025	0.003
**HMGA1**	high mobility group AT-hook 1	0.61	0.73	0.026	0.018
**HMGCR**	3-hydroxy-3-methylglutaryl-CoA reductase	0.72	0.44	0.048	0.002
**HNMT**	histamine N-methyltransferase	**2.29**	**2.58**	0.011	0.013
**HOMER2**	homer homolog 2 (Drosophila)	0.50	0.34	0.045	0.025
**HOMER3**	homer homolog 3 (Drosophila)	**2.34**	**2.66**	0.026	0.008
**HOOK1**	hook homolog 1 (Drosophila)	0.36	0.32	0.031	0.024
**HOXA10 /// HOXA9**	homeobox A10 /// homeobox A9	**1.89**	**1.83**	0.013	0.027

**HP1BP3**	heterochromatin protein 1, binding protein 3	0.57	0.56	0.030	0.042
**HPDL**	4-hydroxyphenylpyruvate dioxygenase-like	0.30	0.30	0.001	0.006
**HSDL2**	hydroxysteroid dehydrogenase like 2	0.64	0.37	0.019	0.011
**ICAM4**	intercellular adhesion molecule 4 (Landsteiner-Wiener blood group)	0.25	0.39	0.004	0.033
**IDH2**	isocitrate dehydrogenase 2 (NADP+), mitochondrial	0.81	0.71	0.014	0.003
**IER2**	immediate early response 2	**1.42**	**1.22**	0.012	0.020
**IFI44L**	interferon-induced protein 44-like	**3.16**	**6.71**	0.020	0.021
**IGHM**	immunoglobulin heavy constant mu	**1.87**	**2.19**	0.039	0.047
**IGLL1**	immunoglobulin lambda-like polypeptide 1	0.78	0.40	0.014	0.026
**IKBIP**	IKBKB interacting protein	**2.46**	**1.48**	0.035	0.046
**IL5RA**	interleukin 5 receptor, alpha	0.53	0.57	0.027	0.007
**ILF3**	interleukin enhancer binding factor 3, 90kDa	0.83	0.75	0.005	0.019
**IRAK1**	interleukin-1 receptor-associated kinase 1	0.68	0.45	0.046	0.000
**IRF1**	interferon regulatory factor 1	**1.72**	**2.16**	0.005	0.004
**IRF2BP2**	interferon regulatory factor 2 binding protein 2	**1.32**	**1.27**	0.027	0.029
**ITGA5**	integrin, alpha 5 (fibronectin receptor, alpha polypeptide)	**1.28**	**2.14**	0.010	0.027
**ITGA6**	integrin, alpha 6	**3.25**	**5.41**	0.028	0.024
**ITGA9**	integrin, alpha 9	**1.72**	**3.39**	0.012	0.020
**ITLN1**	intelectin 1 (galactofuranose binding)	0.08	0.13	0.003	0.005
**JAM3**	junctional adhesion molecule 3	**1.24**	**3.03**	0.029	0.034
**JAZF1**	JAZF zinc finger 1	0.49	0.38	0.037	0.002
**JUP**	junction plakoglobin	**1.43**	**2.33**	0.003	0.026
**KCNH2**	potassium voltage-gated channel, subfamily H (eag-related), member 2	0.20	0.31	0.005	0.007
**KCNK5**	potassium channel, subfamily K, member 5	0.53	0.49	0.030	0.046
**KDM1B**	lysine (K)-specific demethylase 1B	**1.44**	**1.79**	0.045	0.035
**KDM3A**	lysine (K)-specific demethylase 3A	**2.11**	**2.02**	0.007	0.001
**KDM5B**	lysine (K)-specific demethylase 5B	**1.80**	**2.05**	0.045	0.014
**KEL**	Kell blood group, metallo-endopeptidase	0.22	0.27	0.009	0.031
**KIAA0040**	KIAA0040	0.56	0.47	0.013	0.013
**KIAA0101**	KIAA0101	0.79	0.66	0.010	0.028
**KIAA0114**	KIAA0114	0.60	0.60	0.028	0.035
**KIAA0125**	KIAA0125	**2.22**	**4.32**	0.012	0.029
**KIF14**	kinesin family member 14	0.82	0.41	0.024	0.014
**KIF22**	kinesin family member 22	0.76	0.52	0.040	0.042
**KIFC1**	kinesin family member C1	0.64	0.54	0.033	0.018
**KLF1**	Kruppel-like factor 1 (erythroid)	0.15	0.23	0.004	0.040
**KLHL24**	kelch-like 24 (Drosophila)	**2.52**	**3.10**	0.017	0.013
**KRT13**	keratin 13	0.09	0.08	0.001	0.003
**L3MBTL2**	l(3)mbt-like 2 (Drosophila)	0.65	0.72	0.038	0.030
**LACTB**	lactamase, beta	**1.32**	**1.35**	0.007	0.040
**LAMP2**	lysosomal-associated membrane protein 2	**2.95**	**1.46**	0.048	0.026
**LAPTM4A**	lysosomal protein transmembrane 4 alpha	**1.66**	**1.82**	0.001	0.000
**LAPTM4B**	lysosomal protein transmembrane 4 beta	**1.28**	**2.08**	0.034	0.032
**LCA5**	Leber congenital amaurosis 5	0.26	0.27	0.006	0.011
**LEPROTL1**	leptin receptor overlapping transcript-like 1	**1.85**	**1.75**	0.028	0.025
**LOC100288525**	hypothetical protein LOC100288525	0.32	0.27	0.007	0.009
**LOC100505633**	hypothetical LOC100505633	0.20	0.14	0.012	0.005
**LOC150776 /// SMPD4**	sphingomyelin phosphodiesterase 4, neutral membrane pseudogene /// sphingomyelin phosphodiesterase 4, neutral membrane (neutral sphingomyelinase-3)	0.75	0.57	0.041	0.019
**LOC158402**	hypothetical protein LOC158402	0.58	0.52	0.007	0.047
**LOC541471 /// NCRNA00152**	hypothetical LOC541471 /// non-protein coding RNA 152	0.61	0.57	0.001	0.045
**LOC728855**	hypothetical LOC728855	**1.31**	**1.99**	0.041	0.023
**LPCAT3**	lysophosphatidylcholine acyltransferase 3	0.44	0.47	0.006	0.002
**LRPAP1**	low density lipoprotein receptor-related protein associated protein 1	0.61	0.39	0.008	0.009

**LRPPRC**	leucine-rich PPR-motif containing	0.69	0.83	0.029	0.045
**LRRC59**	leucine rich repeat containing 59	0.70	0.73	0.018	0.016
**LSM11**	LSM11, U7 small nuclear RNA associated	0.64	0.66	0.036	0.040
**LSM4**	LSM4 homolog, U6 small nuclear RNA associated (S, cerevisiae)	0.78	0.73	0.047	0.036
**LSS**	lanosterol synthase (2,3-oxidosqualene-lanosterol cyclase)	0.72	0.66	0.039	0.028
**LTBP1**	latent transforming growth factor beta binding protein 1	0.33	0.38	0.014	0.017
**MACF1**	microtubule-actin crosslinking factor 1	**1.70**	**1.92**	0.006	0.021
**MAF**	v-maf musculoaponeurotic fibrosarcoma oncogene homolog (avian)	**1.62**	**2.21**	0.041	0.006
**MAFK**	v-maf musculoaponeurotic fibrosarcoma oncogene homolog K (avian)	**1.93**	**1.57**	0.017	0.039
**MAGEE1**	melanoma antigen family E, 1	0.69	0.64	0.007	0.005
**MAN2A1**	mannosidase, alpha, class 2A, member 1	0.69	0.33	0.043	0.018
**MAP2K6**	mitogen-activated protein kinase kinase 6	0.60	0.49	0.025	0.018
**MAPK6**	mitogen-activated protein kinase 6	**1.54**	**1.57**	0.010	0.001
**MAST4**	microtubule associated serine/threonine kinase family member 4	**1.41**	**3.45**	0.008	0.030
**MBOAT2**	membrane bound O-acyltransferase domain containing 2	0.41	0.52	0.007	0.027
**MCFD2**	multiple coagulation factor deficiency 2	0.63	0.26	0.049	0.006
**MCM10**	minichromosome maintenance complex component 10	0.56	0.61	0.036	0.016
**MCM2**	minichromosome maintenance complex component 2	0.76	0.55	0.034	0.022
**MCM3**	minichromosome maintenance complex component 3	0.74	0.26	0.027	0.002
**MCM7**	minichromosome maintenance complex component 7	0.71	0.60	0.030	0.009
**MCTP1**	multiple C2 domains, transmembrane 1	**1.42**	**2.07**	0.005	0.005
**MED16**	mediator complex subunit 16	0.61	0.61	0.028	0.018
**MEF2C**	myocyte enhancer factor 2C	**1.50**	**2.08**	0.019	0.011
**MEIS1**	Meis homeobox 1	**1.63**	**4.24**	0.007	0.002
**METTL13**	methyltransferase like 13	0.71	0.65	0.025	0.002
**METTL2A /// METTL2B**	methyltransferase like 2A /// methyltransferase like 2B	0.74	0.66	0.012	0.016
**MGA**	MAX gene associated	**1.66**	**1.67**	0.022	0.022
**MGAT3**	mannosyl (beta-1,4-)-glycoprotein beta-1,4-N-acetylglucosaminyltransferase	0.55	0.66	0.034	0.026
**MGC21881**	hypothetical locus MGC21881	0.49	0.45	0.003	0.009
**MGST3**	microsomal glutathione S-transferase 3	0.63	0.50	0.029	0.009
**MICAL2**	microtubule associated monoxygenase, calponin and LIM domain containing 2	0.39	0.48	0.006	0.036
**MKI67**	antigen identified by monoclonal antibody Ki-67	0.69	0.40	0.045	0.009
**MKRN1**	makorin ring finger protein 1	**1.26**	**1.34**	0.034	0.008
**MLF1IP**	MLF1 interacting protein	0.61	0.58	0.029	0.016
**MLKL**	mixed lineage kinase domain-like	**1.50**	**2.35**	0.029	0.034
**MPP1**	membrane protein, palmitoylated 1, 55kDa	0.58	0.72	0.016	0.020
**MRM1**	mitochondrial rRNA methyltransferase 1 homolog (S, cerevisiae)	0.60	0.76	0.011	0.031
**MRPL12**	mitochondrial ribosomal protein L12	0.62	0.79	0.001	0.013
**MRPL27**	mitochondrial ribosomal protein L27	0.73	0.75	0.024	0.018
**MRPL35**	mitochondrial ribosomal protein L35	0.62	0.58	0.002	0.012
**MRPL36**	mitochondrial ribosomal protein L36	0.77	0.76	0.008	0.039
**MRPL37**	mitochondrial ribosomal protein L37	0.77	0.73	0.006	0.017
**MRPS17 /// ZNF713**	mitochondrial ribosomal protein S17 /// zinc finger protein 713	0.68	0.75	0.000	0.042
**MRTO4**	mRNA turnover 4 homolog (S, cerevisiae)	0.73	0.78	0.006	0.029
**MSI2**	musashi homolog 2 (Drosophila)	**1.48**	**2.33**	0.037	0.012
**MT1F**	metallothionein 1F	0.56	0.63	0.005	0.011
**MTF2**	metal response element binding transcription factor 2	**1.59**	**1.71**	0.010	0.015
**MTHFD1**	methylenetetrahydrofolate dehydrogenase (NADP+ dependent) 1, methenyltetrahydrofolate cyclohydrolase, formyltetrahydrofolate synthetase	0.76	0.70	0.010	0.004
**MTHFR**	methylenetetrahydrofolate reductase (NAD(P)H)	0.61	0.56	0.014	0.013

**MTMR12**	myotubularin related protein 12	0.64	0.59	0.015	0.022
**MTMR14**	myotubularin related protein 14	0.81	0.70	0.043	0.040
**MXD1**	MAX dimerization protein 1	0.36	0.43	0.019	0.018
**MYBL2**	v-myb myeloblastosis viral oncogene homolog (avian)-like 2	0.69	0.52	0.003	0.017
**MYH10**	myosin, heavy chain 10, non-muscle	0.27	0.45	0.005	0.028
**MYL4**	myosin, light chain 4, alkali; atrial, embryonic	0.42	0.39	0.011	0.015
**MYO19**	myosin XIX	0.74	0.76	0.050	0.010
**NASP**	nuclear autoantigenic sperm protein (histone-binding)	0.79	0.64	0.011	0.018
**NBAS**	neuroblastoma amplified sequence	0.71	0.68	0.005	0.017
**NCAPH**	non-SMC condensin I complex, subunit H	0.71	0.45	0.001	0.016
**NCOA1**	nuclear receptor coactivator 1	0.66	0.73	0.012	0.027
**NCOA3**	nuclear receptor coactivator 3	**1.78**	**1.56**	0.013	0.020
**NCRNA00275**	non-protein coding RNA 275	**1.29**	**1.94**	0.039	0.023
**NDUFB6**	NADH dehydrogenase (ubiquinone) 1 beta subcomplex, 6, 17kDa	0.74	0.75	0.008	0.044
**NDUFB9**	NADH dehydrogenase (ubiquinone) 1 beta subcomplex, 9, 22kDa	0.80	0.82	0.024	0.048
**NDUFS7**	NADH dehydrogenase (ubiquinone) Fe-S protein 7, 20kDa (NADH-coenzyme Q reductase)	0.67	0.59	0.035	0.005
**NEK2**	NIMA (never in mitosis gene a)-related kinase 2	0.82	0.33	0.041	0.005
**NEK6**	NIMA (never in mitosis gene a)-related kinase 6	**1.52**	**2.19**	0.013	0.015
**NFIA**	nuclear factor I/A	0.35	0.40	0.009	0.013
**NIPA2**	non imprinted in Prader-Willi/Angelman syndrome 2	0.61	0.62	0.005	0.007
**NOG**	noggin	**4.84**	**11.16**	0.012	0.004
**NOTCH1**	notch 1	**1.59**	**1.24**	0.023	0.001
**NPL**	N-acetylneuraminate pyruvate lyase (dihydrodipicolinate synthase)	0.38	0.45	0.016	0.039
**NPRL3**	nitrogen permease regulator-like 3 (S, cerevisiae)	0.48	0.56	0.006	0.018
**NRIP1**	nuclear receptor interacting protein 1	**1.66**	**2.35**	0.033	0.020
**NT5DC2**	5-nucleotidase domain containing 2	0.68	0.58	0.032	0.006
**NUDT21**	nudix (nucleoside diphosphate linked moiety X)-type motif 21	0.67	0.49	0.046	0.050
**NUP62**	nucleoporin 62kDa	0.79	0.82	0.029	0.019
**OAT**	ornithine aminotransferase	0.68	0.63	0.045	0.030
**OBFC2A**	oligonucleotide/oligosaccharide-binding fold containing 2A	**1.85**	**1.73**	0.010	0.015
**OGFOD2**	2-oxoglutarate and iron-dependent oxygenase domain containing 2	0.71	0.70	0.043	0.015
**OGT**	O-linked N-acetylglucosamine (GlcNAc) transferase (UDP-N-acetylglucosamine:polypeptide-N-acetylglucosaminyl transferase)	**1.52**	**1.52**	0.028	0.025
**OSTM1**	osteopetrosis associated transmembrane protein 1	**2.01**	**2.30**	0.047	0.020
**P2RY8**	purinergic receptor P2Y, G-protein coupled, 8	**1.49**	**2.30**	0.004	0.007
**P4HB**	prolyl 4-hydroxylase, beta polypeptide	0.76	0.73	0.034	0.038
**PA2G4**	proliferation-associated 2G4, 38kDa	0.67	0.68	0.002	0.008
**PAFAH1B3**	platelet-activating factor acetylhydrolase 1b, catalytic subunit 3 (29kDa)	0.55	0.65	0.001	0.028
**PAIP2**	poly(A) binding protein interacting protein 2	**1.23**	**1.39**	0.006	0.032
**PAQR7**	progestin and adipoQ receptor family member VII	**1.42**	**1.69**	0.012	0.002
**PARP14**	poly (ADP-ribose) polymerase family, member 14	**1.52**	**1.37**	0.007	0.037
**PBK**	PDZ binding kinase	0.26	0.34	0.003	0.003
**PCCB**	propionyl CoA carboxylase, beta polypeptide	0.47	0.68	0.007	0.020
**PCGF5**	polycomb group ring finger 5	0.47	0.47	0.037	0.035
**PCMTD1**	protein-L-isoaspartate (D-aspartate) O-methyltransferase domain containing 1	**1.48**	**1.62**	0.009	0.016
**PCNX**	pecanex homolog (Drosophila)	**2.50**	**1.70**	0.049	0.039
**PDK3**	pyruvate dehydrogenase kinase, isozyme 3	0.53	0.68	0.004	0.025
**PDLIM1**	PDZ and LIM domain 1	0.71	0.58	0.023	0.031
**PDLIM5**	PDZ and LIM domain 5	**1.34**	**2.22**	0.035	0.022
**PDSS1**	prenyl (decaprenyl) diphosphate synthase, subunit 1	0.79	0.62	0.030	0.006
**PECAM1**	platelet/endothelial cell adhesion molecule	**2.11**	**3.17**	0.011	0.004

**PECI**	peroxisomal D3,D2-enoyl-CoA isomerase	0.62	0.28	0.010	0.011
**PFDN6**	prefoldin subunit 6	0.72	0.73	0.042	0.031
**PGP**	phosphoglycolate phosphatase	0.58	0.53	0.021	0.018
**PHF10**	PHD finger protein 10	0.82	0.73	0.032	0.010
**PHGDH**	phosphoglycerate dehydrogenase	0.62	0.80	0.037	0.037
**PHTF1**	putative homeodomain transcription factor 1	**2.32**	**1.82**	0.032	0.012
**PIGC**	phosphatidylinositol glycan anchor biosynthesis, class C	0.59	0.79	0.008	0.027
**PIGH**	phosphatidylinositol glycan anchor biosynthesis, class H	0.44	0.62	0.000	0.033
**PIGV**	phosphatidylinositol glycan anchor biosynthesis, class V	0.48	0.74	0.038	0.017
**PIK3AP1**	phosphoinositide-3-kinase adaptor protein 1	**1.50**	**1.38**	0.001	0.004
**PIN1**	peptidylprolyl cis/trans isomerase, NIMA-interacting 1	0.72	0.73	0.035	0.033
**PIP4K2A**	phosphatidylinositol-5-phosphate 4-kinase, type II, alpha	0.74	0.65	0.018	0.019
**PISD**	phosphatidylserine decarboxylase	0.80	0.75	0.017	0.015
**PITPNB**	phosphatidylinositol transfer protein, beta	0.79	0.55	0.026	0.006
**PKLR**	pyruvate kinase, liver and RBC	0.12	0.10	0.000	0.001
**PLA2G12A**	phospholipase A2, group XIIA	0.70	0.43	0.042	0.018
**PLAU**	plasminogen activator, urokinase	**1.95**	**2.50**	0.008	0.019
**PLCB4**	phospholipase C, beta 4	**1.73**	**3.93**	0.001	0.042
**PLD1**	phospholipase D1, phosphatidylcholine-specific	0.52	0.72	0.015	0.021
**PLEKHA5**	pleckstrin homology domain containing, family A member 5	**1.81**	**1.72**	0.040	0.011
**PLK1**	polo-like kinase 1	0.67	0.43	0.034	0.023
**PNMT**	phenylethanolamine N-methyltransferase	0.22	0.35	0.002	0.018
**PNPO**	pyridoxamine 5-phosphate oxidase	0.42	0.51	0.005	0.001
**PNRC1**	proline-rich nuclear receptor coactivator 1	**1.55**	**1.66**	0.002	0.031
**POGK**	pogo transposable element with KRAB domain	**1.50**	**1.31**	0.011	0.023
**POLD1**	polymerase (DNA directed), delta 1, catalytic subunit 125kDa	0.72	0.65	0.012	0.016
**POLD2**	polymerase (DNA directed), delta 2, regulatory subunit 50kDa	0.75	0.60	0.014	0.013
**POLDIP2**	polymerase (DNA-directed), delta interacting protein 2	0.75	0.73	0.033	0.011
**POLR2C**	polymerase (RNA) II (DNA directed) polypeptide C, 33kDa	0.62	0.69	0.002	0.009
**POLR2F**	polymerase (RNA) II (DNA directed) polypeptide F	0.70	0.76	0.044	0.014
**POLR2L**	polymerase (RNA) II (DNA directed) polypeptide L, 7,6kDa	0.78	0.76	0.027	0.030
**POP7**	processing of precursor 7, ribonuclease P/MRP subunit (S, cerevisiae)	0.62	0.61	0.021	0.017
**PPCS**	phosphopantothenoylcysteine synthetase	**1.85**	**1.55**	0.011	0.017
**PPIL2**	peptidylprolyl isomerase (cyclophilin)-like 2	0.72	0.75	0.019	0.022
**PPM1A**	protein phosphatase, Mg2+/Mn2+ dependent, 1A	0.69	0.64	0.012	0.022
**PPME1**	protein phosphatase methylesterase 1	0.58	0.65	0.030	0.034
**PPOX**	protoporphyrinogen oxidase	0.32	0.33	0.001	0.016
**PPP1R3D**	protein phosphatase 1, regulatory (inhibitor) subunit 3D	**1.58**	**1.53**	0.029	0.021
**PPT2**	palmitoyl-protein thioesterase 2	0.48	0.39	0.011	0.027
**PRC1**	protein regulator of cytokinesis 1	0.69	0.53	0.001	0.009
**PRDX2**	peroxiredoxin 2	0.30	0.29	0.002	0.014
**PRICKLE1**	prickle homolog 1 (Drosophila)	**2.71**	**1.78**	0.027	0.009
**PRKAB1**	protein kinase, AMP-activated, beta 1 non-catalytic subunit	0.40	0.80	0.012	0.001
**PRKAG2**	protein kinase, AMP-activated, gamma 2 non-catalytic subunit	**1.95**	**1.48**	0.028	0.033
**PRKAR2B**	protein kinase, cAMP-dependent, regulatory, type II, beta	0.27	0.33	0.033	0.040
**PRKCH**	protein kinase C, eta	**1.86**	**3.63**	0.036	0.002
**PROM1**	prominin 1	**2.67**	**8.50**	0.044	0.026
**PRPF31**	PRP31 pre-mRNA processing factor 31 homolog (S, cerevisiae)	0.80	0.81	0.011	0.044
**PRPS1**	phosphoribosyl pyrophosphate synthetase 1	0.68	0.77	0.043	0.043
**PSAT1**	phosphoserine aminotransferase 1	0.79	0.56	0.012	0.015
**PSMD11**	proteasome (prosome, macropain) 26S subunit, non-ATPase, 11	0.83	0.34	0.032	0.006
**PSMD13**	proteasome (prosome, macropain) 26S subunit, non-ATPase, 13	0.75	0.67	0.028	0.039
**PSMD8**	proteasome (prosome, macropain) 26S subunit, non-ATPase, 8	0.62	0.75	0.001	0.022
**PSME3**	proteasome (prosome, macropain) activator subunit 3 (PA28 gamma; Ki)	0.63	0.70	0.034	0.008

**PTEN**	phosphatase and tensin homolog	**1.48**	**1.35**	0.020	0.034
**PTEN /// PTENP1**	phosphatase and tensin homolog /// phosphatase and tensin homolog pseudogene 1	**1.62**	**1.30**	0.003	0.015
**PTPN18**	protein tyrosine phosphatase, non-receptor type 18 (brain-derived)	0.74	0.63	0.043	0.029
**PUS1**	pseudouridylate synthase 1	0.73	0.75	0.018	0.047
**PVT1**	Pvt1 oncogene (non-protein coding)	0.07	0.30	0.004	0.035
**PYGL**	phosphorylase, glycogen, liver	**1.58**	**1.83**	0.049	0.002
**RAB8B**	RAB8B, member RAS oncogene family	**1.55**	**1.89**	0.049	0.035
**RABGAP1L**	RAB GTPase activating protein 1-like	0.64	0.59	0.026	0.000
**RACGAP1**	Rac GTPase activating protein 1	0.51	0.60	0.000	0.015
**RAD23A**	RAD23 homolog A (S, cerevisiae)	0.53	0.57	0.030	0.023
**RANBP1**	RAN binding protein 1	0.78	0.79	0.012	0.022
**RARRES3**	retinoic acid receptor responder (tazarotene induced) 3	**1.92**	**4.00**	0.014	0.027
**RAVER1**	ribonucleoprotein, PTB-binding 1	0.58	0.55	0.012	0.005
**RBM22**	RNA binding motif protein 22	**1.27**	**1.24**	0.006	0.029
**RBMS1**	RNA binding motif, single stranded interacting protein 1	**1.33**	**1.76**	0.024	0.010
**RCCD1**	RCC1 domain containing 1	0.73	0.45	0.010	0.001
**RDBP**	RD RNA binding protein	0.71	0.63	0.007	0.020
**RECK**	reversion-inducing-cysteine-rich protein with kazal motifs	**3.43**	**5.35**	0.019	0.001
**REXO2**	REX2, RNA exonuclease 2 homolog (S, cerevisiae)	0.37	0.38	0.000	0.004
**RFC2**	replication factor C (activator 1) 2, 40kDa	0.77	0.71	0.038	0.001
**RFC4**	replication factor C (activator 1) 4, 37kDa	0.76	0.54	0.011	0.010
**RFESD**	Rieske (Fe-S) domain containing	0.22	0.19	0.010	0.008
**RHAG**	Rh-associated glycoprotein	0.07	0.17	0.004	0.022
**RHOBTB1**	Rho-related BTB domain containing 1	**1.85**	**1.84**	0.012	0.015
**RILP**	Rab interacting lysosomal protein	0.20	0.58	0.001	0.005
**RILPL2**	Rab interacting lysosomal protein-like 2	**1.42**	**1.20**	0.004	0.012
**RIT1**	Ras-like without CAAX 1	**1.66**	**1.66**	0.014	0.026
**RMND5B**	required for meiotic nuclear division 5 homolog B (S, cerevisiae)	0.27	0.73	0.002	0.008
**RNASE1**	ribonuclease, RNase A family, 1 (pancreatic)	0.18	0.18	0.002	0.010
**RNASE4**	ribonuclease, RNase A family, 4	0.24	0.44	0.020	0.000
**RNASEH2A**	ribonuclease H2, subunit A	0.68	0.53	0.018	0.015
**RNF113A**	ring finger protein 113A	**1.30**	**1.45**	0.038	0.045
**RNF121**	ring finger protein 121	0.54	0.68	0.028	0.009
**RNF123**	ring finger protein 123	0.56	0.51	0.025	0.017
**RNF126**	ring finger protein 126	0.38	0.80	0.002	0.038
**RNF149**	ring finger protein 149	**1.47**	**1.44**	0.000	0.006
**RNF26**	ring finger protein 26	0.48	0.47	0.045	0.011
**RNF34**	ring finger protein 34	0.61	0.45	0.028	0.020
**RPP21 /// TRIM39 /// TRIM39R**	ribonuclease P/MRP 21kDa subunit /// tripartite motif-containing 39 /// TRIM39-like protein	0.71	0.81	0.038	0.010
**RPRD1A**	regulation of nuclear pre-mRNA domain containing 1A	0.43	0.51	0.031	0.030
**RPS6KL1**	ribosomal protein S6 kinase-like 1	0.48	0.44	0.006	0.002
**RRAGD**	Ras-related GTP binding D	**1.45**	**1.85**	0.026	0.015
**RTP4**	receptor (chemosensory) transporter protein 4	**2.16**	**4.76**	0.017	0.004
**RUVBL2**	RuvB-like 2 (E, coli)	0.67	0.68	0.019	0.026
**RYR3**	ryanodine receptor 3	0.38	0.57	0.019	0.033
**SAMM50**	sorting and assembly machinery component 50 homolog (S, cerevisiae)	0.54	0.69	0.003	0.004
**SAP130**	Sin3A-associated protein, 130kDa	0.61	0.64	0.017	0.015
**SAP30BP**	SAP30 binding protein	0.73	0.76	0.030	0.005
**SARNP**	SAP domain containing ribonucleoprotein	0.70	0.67	0.011	0.032
**SARS**	seryl-tRNA synthetase	0.73	0.72	0.037	0.013
**SART1**	squamous cell carcinoma antigen recognized by T cells	0.75	0.80	0.033	0.019
**SATB1**	SATB homeobox 1	**1.42**	**1.96**	0.048	0.034
**SBF2**	SET binding factor 2	**1.88**	**2.93**	0.031	0.041

**SCPEP1**	serine carboxypeptidase 1	**1.52**	**1.76**	0.002	0.037
**SDHA**	succinate dehydrogenase complex, subunit A, flavoprotein (Fp)	0.70	0.71	0.043	0.003
**SEC14L2**	SEC14-like 2 (S, cerevisiae)	0.59	0.70	0.034	0.025
**SEC22C**	SEC22 vesicle trafficking protein homolog C (S, cerevisiae)	0.72	0.58	0.013	0.035
**SEC62**	SEC62 homolog (S, cerevisiae)	**1.41**	**1.35**	0.012	0.001
**SEL1L3**	sel-1 suppressor of lin-12-like 3 (C, elegans)	**2.31**	**1.79**	0.005	0.017
**SELENBP1**	selenium binding protein 1	0.05	0.09	0.001	0.002
**SEMA4D**	sema domain, immunoglobulin domain (Ig), transmembrane domain (TM) and short cytoplasmic domain, (semaphorin) 4D	**1.94**	**2.67**	0.042	0.015
**SEPP1**	selenoprotein P, plasma, 1	**6.08**	**3.52**	0.009	0.004
**SERPINB1**	serpin peptidase inhibitor, clade B (ovalbumin), member 1	**1.45**	**1.58**	0.026	0.012
**SESN1**	sestrin 1	**1.73**	**1.89**	0.001	0.004
**SETD8**	SET domain containing (lysine methyltransferase) 8	0.69	0.57	0.017	0.006
**SFXN1**	sideroflexin 1	0.67	0.45	0.030	0.014
**SGPL1**	sphingosine-1-phosphate lyase 1	**1.95**	**1.26**	0.016	0.036
**SHB**	Src homology 2 domain containing adaptor protein B	**1.41**	**1.89**	0.046	0.009
**SHKBP1**	SH3KBP1 binding protein 1	0.49	0.66	0.016	0.006
**SHMT2**	serine hydroxymethyltransferase 2 (mitochondrial)	0.73	0.75	0.024	0.013
**SIAH2**	seven in absentia homolog 2 (Drosophila)	0.66	0.78	0.013	0.001
**SIGMAR1**	sigma non-opioid intracellular receptor 1	0.50	0.60	0.009	0.009
**SLC12A6**	solute carrier family 12 (potassium/chloride transporters), member 6	0.48	0.53	0.014	0.014
**SLC16A9**	solute carrier family 16, member 9 (monocarboxylic acid transporter 9)	0.19	0.16	0.003	0.011
**SLC17A5**	solute carrier family 17 (anion/sugar transporter), member 5	**1.50**	**1.58**	0.017	0.017
**SLC22A23**	solute carrier family 22, member 23	0.29	0.30	0.010	0.011
**SLC25A1**	solute carrier family 25 (mitochondrial carrier; citrate transporter), member 1	0.58	0.61	0.008	0.012
**SLC25A11**	solute carrier family 25 (mitochondrial carrier; oxoglutarate carrier), member 11	0.72	0.78	0.007	0.004
**SLC25A12**	solute carrier family 25 (mitochondrial carrier, Aralar), member 12	**3.21**	**1.31**	0.025	0.035
**SLC25A17**	solute carrier family 25 (mitochondrial carrier; peroxisomal membrane protein, 34kDa), member 17	0.62	0.47	0.041	0.009
**SLC25A21**	solute carrier family 25 (mitochondrial oxodicarboxylate carrier), member 21	0.11	0.05	0.001	0.006
**SLC25A28**	solute carrier family 25, member 28	0.78	0.75	0.032	0.019
**SLC25A37**	solute carrier family 25, member 37	0.47	0.41	0.017	0.013
**SLC25A39**	solute carrier family 25, member 39	0.61	0.43	0.004	0.006
**SLC26A2**	solute carrier family 26 (sulfate transporter), member 2	0.45	0.33	0.010	0.011
**SLC29A1**	solute carrier family 29 (nucleoside transporters), member 1	0.55	0.72	0.019	0.028
**SLC29A2**	solute carrier family 29 (nucleoside transporters), member 2	0.68	0.77	0.023	0.026
**SLC31A1**	solute carrier family 31 (copper transporters), member 1	0.73	0.68	0.011	0.010
**SLC35A4**	solute carrier family 35, member A4	0.52	0.64	0.018	0.015
**SLC35D2**	solute carrier family 35, member D2	**1.96**	**3.62**	0.015	0.005
**SLC37A4**	solute carrier family 37 (glucose-6-phosphate transporter), member 4	0.49	0.48	0.011	0.017
**SLC38A5**	solute carrier family 38, member 5	0.29	0.33	0.000	0.015
**SLC41A2**	solute carrier family 41, member 2	0.44	0.25	0.009	0.015
**SLC43A3**	solute carrier family 43, member 3	0.56	0.48	0.002	0.014
**SLC9A3R1**	solute carrier family 9 (sodium/hydrogen exchanger), member 3 regulator 1	**1.30**	**1.52**	0.010	0.006
**SLX1A /// SLX1B**	SLX1 structure-specific endonuclease subunit homolog A (S, cerevisiae) /// SLX1 structure-specific endonuclease subunit homolog B (S, cerevisiae)	0.62	0.60	0.013	0.019
**SMURF2**	SMAD specific E3 ubiquitin protein ligase 2	**1.76**	**1.73**	0.032	0.038
**SNHG1**	small nucleolar RNA host gene 1 (non-protein coding)	0.83	0.75	0.041	0.028
**SNRK**	SNF related kinase	**1.87**	**1.88**	0.033	0.009
**SNRNP27**	small nuclear ribonucleoprotein 27kDa (U4/U6,U5)	0.67	0.71	0.008	0.044
**SNX9**	sorting nexin 9	0.66	0.74	0.048	0.001

**SORBS1**	sorbin and SH3 domain containing 1	0.26	0.29	0.016	0.035
**SORL1**	sortilin-related receptor, L(DLR class) A repeats-containing	**2.36**	**2.97**	0.003	0.016
**SOX4**	SRY (sex determining region Y)-box 4	**1.52**	**2.34**	0.007	0.008
**SP1**	Sp1 transcription factor	**1.78**	**1.25**	0.017	0.033
**SPAG5**	sperm associated antigen 5	0.75	0.59	0.015	0.029
**SPARC**	secreted protein, acidic, cysteine-rich (osteonectin)	**2.24**	**4.54**	0.003	0.021
**SPCS3**	signal peptidase complex subunit 3 homolog (S, cerevisiae)	**1.23**	**1.28**	0.013	0.003
**SPIN1**	spindlin 1	**1.28**	**1.63**	0.013	0.015
**SPNS1**	spinster homolog 1 (Drosophila)	0.73	0.59	0.045	0.004
**SPOP**	speckle-type POZ protein	**1.32**	**1.35**	0.001	0.001
**SPTA1**	spectrin, alpha, erythrocytic 1 (elliptocytosis 2)	0.19	0.12	0.023	0.019
**SPTBN1**	spectrin, beta, non-erythrocytic 1	**2.07**	**2.81**	0.029	0.018
**SPTLC2**	Serine palmitoyltransferase, long chain base subunit 2	**1.56**	**1.28**	0.038	0.036
**SRBD1**	S1 RNA binding domain 1	**1.82**	**1.88**	0.024	0.008
**SRD5A1**	steroid-5-alpha-reductase, alpha polypeptide 1 (3-oxo-5 alpha-steroid delta 4-dehydrogenase alpha 1)	0.69	0.51	0.038	0.022
**SRP14**	signal recognition particle 14kDa (homologous Alu RNA binding protein)	**1.25**	**1.23**	0.014	0.029
**SRXN1**	sulfiredoxin 1	0.42	0.66	0.011	0.049
**SSRP1**	structure specific recognition protein 1	0.79	0.58	0.017	0.005
**SSX2IP**	synovial sarcoma, X breakpoint 2 interacting protein	0.61	0.66	0.034	0.021
**ST3GAL6**	ST3 beta-galactoside alpha-2,3-sialyltransferase 6	**2.74**	**1.76**	0.006	0.003
**STAP1**	signal transducing adaptor family member 1	**1.70**	**2.97**	0.048	0.011
**STK35**	serine/threonine kinase 35	**1.74**	**1.58**	0.019	0.021
**STRA13**	stimulated by retinoic acid 13 homolog (mouse)	0.68	0.70	0.018	0.016
**STUB1**	STIP1 homology and U-box containing protein 1, E3 ubiquitin protein ligase	0.66	0.79	0.019	0.006
**STX2**	syntaxin 2	0.59	0.32	0.044	0.014
**SUPT6H**	suppressor of Ty 6 homolog (S, cerevisiae)	0.75	0.67	0.006	0.029
**SWAP70**	SWAP switching B-cell complex 70kDa subunit	**1.62**	**1.47**	0.000	0.042
**SYNGR2**	synaptogyrin 2	0.75	0.74	0.011	0.000
**SYNJ2BP**	synaptojanin 2 binding protein	**2.35**	**2.40**	0.029	0.007
**TAB2**	TGF-beta activated kinase 1/MAP3K7 binding protein 2	**1.34**	**2.23**	0.020	0.024
**TATDN3**	TatD DNase domain containing 3	**1.61**	**1.66**	0.021	0.011
**TBC1D2B**	TBC1 domain family, member 2B	**1.63**	**1.55**	0.012	0.010
**TBCD**	tubulin folding cofactor D	0.78	0.83	0.023	0.014
**TBL1X**	transducin (beta)-like 1X-linked	0.51	0.71	0.009	0.020
**TBL3**	transducin (beta)-like 3	0.78	0.72	0.027	0.002
**TBXAS1**	thromboxane A synthase 1 (platelet)	**1.47**	**1.75**	0.045	0.013
**TCEAL4**	transcription elongation factor A (SII)-like 4	**1.33**	**1.68**	0.050	0.033
**TCEB2**	transcription elongation factor B (SIII), polypeptide 2 (18kDa, elongin B)	0.74	0.57	0.013	0.015
**TCF7L2**	transcription factor 7-like 2 (T-cell specific, HMG-box)	**1.67**	**2.91**	0.036	0.015
**TELO2**	TEL2, telomere maintenance 2, homolog (S, cerevisiae)	0.78	0.80	0.032	0.043
**TEX261**	testis expressed 261	0.76	0.62	0.045	0.002
**TFEC**	transcription factor EC	**2.18**	**2.36**	0.001	0.009
**TFR2**	transferrin receptor 2	0.21	0.34	0.006	0.009
**TFRC**	transferrin receptor (p90, CD71)	0.64	0.60	0.026	0.034
**TH1L**	TH1-like (Drosophila)	0.69	0.79	0.007	0.006
**TIMELESS**	timeless homolog (Drosophila)	0.80	0.66	0.042	0.016
**TIMM50**	translocase of inner mitochondrial membrane 50 homolog (S, cerevisiae)	0.64	0.62	0.019	0.022
**TIMM8B**	translocase of inner mitochondrial membrane 8 homolog B (yeast)	0.76	0.80	0.020	0.033
**TIMP2**	TIMP metallopeptidase inhibitor 2	**2.13**	**1.60**	0.008	0.046
**TK1**	thymidine kinase 1, soluble	0.78	0.46	0.049	0.020
**TLE4**	transducin-like enhancer of split 4 (E(sp1) homolog, Drosophila)	**1.71**	**1.46**	0.036	0.030
**TM7SF3**	transmembrane 7 superfamily member 3	0.79	0.30	0.036	0.006
**TMCO6**	transmembrane and coiled-coil domains 6	0.65	0.66	0.027	0.026

**TMED5**	transmembrane emp24 protein transport domain containing 5	**1.56**	**1.66**	0.021	0.008
**TMEM138**	transmembrane protein 138	0.72	0.52	0.021	0.003
**TMEM141**	transmembrane protein 141	0.41	0.49	0.001	0.009
**TMEM14B**	transmembrane protein 14B	0.37	0.66	0.002	0.005
**TMEM14B /// TMEM14C**	transmembrane protein 14B /// transmembrane protein 14C	0.54	0.75	0.000	0.049
**TMEM14C**	transmembrane protein 14C	0.52	0.63	0.001	0.039
**TMEM156**	transmembrane protein 156	**2.23**	**1.29**	0.001	0.031
**TMEM165**	transmembrane protein 165	0.73	0.63	0.008	0.000
**TMEM177**	transmembrane protein 177	0.67	0.80	0.018	0.003
**TMEM19**	transmembrane protein 19	0.56	0.33	0.029	0.014
**TMEM30A**	transmembrane protein 30A	**2.12**	**2.09**	0.045	0.017
**TMEM56**	transmembrane protein 56	0.13	0.06	0.008	0.000
**TMEM97**	transmembrane protein 97	0.56	0.47	0.016	0.032
**TMF1**	TATA element modulatory factor 1	0.54	0.59	0.017	0.029
**TMOD1**	tropomodulin 1	0.32	0.52	0.027	0.030
**TNFRSF10D**	tumor necrosis factor receptor superfamily, member 10d, decoy with truncated death domain	**2.13**	**3.63**	0.006	0.000
**TNFRSF19**	tumor necrosis factor receptor superfamily, member 19	0.26	0.37	0.029	0.042
**TNFRSF1A**	tumor necrosis factor receptor superfamily, member 1A	**1.64**	**1.93**	0.046	0.020
**TNFSF4**	tumor necrosis factor (ligand) superfamily, member 4	**3.17**	**2.91**	0.014	0.027
**TOMM40**	translocase of outer mitochondrial membrane 40 homolog (yeast)	0.69	0.77	0.044	0.011
**TOP3A**	topoisomerase (DNA) III alpha	0.71	0.68	0.021	0.018
**TP53**	tumor protein p53	0.75	0.78	0.026	0.011
**TP53INP1**	tumor protein p53 inducible nuclear protein 1	**2.68**	**2.86**	0.010	0.013
**TPM1**	tropomyosin 1 (alpha)	0.29	0.51	0.013	0.028
**TPP2**	tripeptidyl peptidase II	**1.53**	**1.53**	0.042	0.029
**TPX2**	TPX2, microtubule-associated, homolog (Xenopus laevis)	0.63	0.60	0.004	0.004
**TRAK2**	trafficking protein, kinesin binding 2	0.48	0.76	0.008	0.037
**TRAP1**	TNF receptor-associated protein 1	0.68	0.73	0.020	0.015
**TROAP**	trophinin associated protein (tastin)	0.69	0.55	0.037	0.016
**TSFM**	Ts translation elongation factor, mitochondrial	0.76	0.75	0.047	0.016
**TSPAN5**	tetraspanin 5	0.29	0.40	0.008	0.021
**TST**	thiosulfate sulfurtransferase (rhodanese)	0.55	0.53	0.013	0.011
**TSTA3**	tissue specific transplantation antigen P35B	0.81	0.74	0.029	0.003
**TTC13**	tetratricopeptide repeat domain 13	0.72	0.50	0.018	0.000
**TTLL11**	tubulin tyrosine ligase-like family, member 11	0.61	0.66	0.013	0.018
**TTLL12**	tubulin tyrosine ligase-like family, member 12	0.64	0.59	0.004	0.005
**TUBB2A**	tubulin, beta 2A	0.31	0.12	0.020	0.006
**TUBB3**	tubulin, beta 3	0.73	0.76	0.042	0.032
**TUBB6**	tubulin, beta 6	0.57	0.51	0.019	0.003
**TUBG1**	tubulin, gamma 1	0.62	0.57	0.024	0.028
**TXLNG**	taxilin gamma	0.72	0.57	0.022	0.013
**U2AF2**	U2 small nuclear RNA auxiliary factor 2	0.66	0.81	0.010	0.024
**UBAC1**	UBA domain containing 1	0.64	0.62	0.020	0.007
**UBE2F**	ubiquitin-conjugating enzyme E2F (putative)	0.71	0.76	0.014	0.002
**UBE2H**	ubiquitin-conjugating enzyme E2H (UBC8 homolog, yeast)	**1.69**	**1.81**	0.013	0.018
**UBE2J2**	ubiquitin-conjugating enzyme E2, J2 (UBC6 homolog, yeast)	0.73	0.81	0.018	0.047
**UBE2L3**	ubiquitin-conjugating enzyme E2L 3	0.65	0.43	0.023	0.013
**UBE3B**	ubiquitin protein ligase E3B	0.45	0.36	0.041	0.007
**UBL7**	ubiquitin-like 7 (bone marrow stromal cell-derived)	0.45	0.55	0.025	0.021
**UCA1**	urothelial cancer associated 1 (non-protein coding)	0.12	0.11	0.000	0.003
**UGGT1**	UDP-glucose glycoprotein glucosyltransferase 1	0.68	0.64	0.008	0.021
**UMPS**	uridine monophosphate synthetase	0.49	0.55	0.025	0.020
**UQCR10**	ubiquinol-cytochrome c reductase, complex III subunit X	0.67	0.76	0.004	0.038
**UROD**	uroporphyrinogen decarboxylase	0.41	0.32	0.018	0.022
**UROS**	uroporphyrinogen III synthase	0.54	0.55	0.002	0.020

**UXS1**	UDP-glucuronate decarboxylase 1	**1.51**	**2.07**	0.041	0.012
**VGLL4**	vestigial like 4 (Drosophila)	**1.27**	**1.40**	0.002	0.035
**VPS25**	vacuolar protein sorting 25 homolog (S, cerevisiae)	0.75	0.81	0.001	0.049
**VPS37B**	vacuolar protein sorting 37 homolog B (S, cerevisiae)	0.49	0.69	0.021	0.045
**WBP11**	WW domain binding protein 11	0.68	0.70	0.024	0.007
**WDR18**	WD repeat domain 18	0.70	0.75	0.007	0.010
**WDR61**	WD repeat domain 61	0.83	0.78	0.013	0.033
**WDR7**	WD repeat domain 7	0.50	0.53	0.030	0.030
**WHSC1**	Wolf-Hirschhorn syndrome candidate 1	0.65	0.70	0.041	0.018
**WHSC1L1**	Wolf-Hirschhorn syndrome candidate 1-like 1	0.83	0.70	0.027	0.040
**WIBG**	within bgcn homolog (Drosophila)	0.66	0.40	0.032	0.034
**WIPF1**	WAS/WASL interacting protein family, member 1	**1.66**	**1.86**	0.030	0.030
**XBP1**	X-box binding protein 1	**1.36**	**1.97**	0.045	0.026
**XK**	X-linked Kx blood group (McLeod syndrome)	0.15	0.21	0.021	0.011
**XPO5**	exportin 5	0.58	0.67	0.005	0.017
**XPR1**	xenotropic and polytropic retrovirus receptor 1	0.59	0.47	0.034	0.037
**XRCC5**	X-ray repair complementing defective repair in Chinese hamster cells 5 (double-strand-break rejoining)	0.78	0.83	0.029	0.008
**YIF1A**	Yip1 interacting factor homolog A (S, cerevisiae)	0.79	0.62	0.040	0.001
**YIF1B**	Yip1 interacting factor homolog B (S, cerevisiae)	0.59	0.62	0.038	0.003
**YPEL5**	yippee-like 5 (Drosophila)	**1.59**	**1.60**	0.016	0.033
**ZDHHC14**	zinc finger, DHHC-type containing 14	0.49	0.37	0.001	0.010
**ZFAND5**	zinc finger, AN1-type domain 5	**1.39**	**1.36**	0.040	0.049
**ZMAT3**	zinc finger, matrin-type 3	**2.11**	**2.50**	0.043	0.027
**ZMYM4**	zinc finger, MYM-type 4	**1.39**	**1.60**	0.023	0.003
**ZMYM5**	zinc finger, MYM-type 5	**1.48**	**1.49**	0.004	0.007
**ZMYND19**	zinc finger, MYND-type containing 19	0.73	0.77	0.002	0.017
**ZNF24**	zinc finger protein 24	**1.49**	**1.47**	0.040	0.037
**ZNF280B**	zinc finger protein 280B	**1.73**	**1.84**	0.039	0.014
**ZNF519**	zinc finger protein 519	0.53	0.55	0.010	0.008
**ZNF655**	zinc finger protein 655	0.63	0.54	0.046	0.009
**ZSCAN16**	zinc finger and SCAN domain containing 16	0.79	0.64	0.033	0.025
**ZSCAN18**	zinc finger and SCAN domain containing 18	**1.21**	**1.81**	0.025	0.036

Supporting Information Table S2
Alteration of metabolic pathways in AK2-deficient cells
(the fold change between shAK2 and shCont is shown for shAK2 #1 and shAK2 #2, upregulated genes are shown in bold type)

OXPHOS genes		#1	#2
**ATPIF1**	ATPase inhibitory factor 1 (**regulator of CxV activity**)	0.49	0.43
**BCS1L**	BCS1-like (**assembly factor of CxIII**)	0.74	0.61
**BOLA3**	bolA homolog 3 (**production of respiratory chain Fe-S clusters**)	0.26	0.69
**COX5A**	cytochrome c oxidase subunit Va (**CxIV subunit**)	0.55	0.57
**GLRX5**	glutaredoxin 5 (**production of respiratory chain Fe-S clusters**)	0.68	0.73
**NDUFB6**	NADH dehydrogenase (ubiquinone) 1 beta 6 (**CxI subunit**)	0.74	0.76
**NDUFB9**	NADH dehydrogenase (ubiquinone) 1 beta 9 **(CxI subunit**)	0.80	0.82
**NDUFS7**	NADH dehydrogenase (ubiquinone) Fe-S protein 7 (**CxI subunit**)	0.67	0.59
**SDHA**	succinate dehydrogenase complex. subunit A. flavoprotein (Fp) (**CxII subunit**)	0.70	0.71
**UQCR10**	ubiquinol-cytochrome c reductase subunit X (**CxIII subunit**)	0.67	0.76

Mitochondrial translation		#1	#2
**CARS2**	cysteinyl-tRNA synthetase 2. mitochondrial (putative)	0.81	0.66
**EARS2**	glutamyl-tRNA synthetase 2. mitochondrial (putative)	0.66	0.67
**GARS**	glycyl-tRNA synthetase	0.68	0.70
**LRPPRC**	leucine-rich PPR-motif containing	0.69	0.83
**MRM1**	mitochondrial rRNA methyltransferase 1 homolog	0.60	0.76
**MRPL12**	mitochondrial ribosomal protein L12	0.62	0.79
**MRPL27**	mitochondrial ribosomal protein L27	0.73	0.76
**MRPL35**	mitochondrial ribosomal protein L35	0.62	0.58
**MRPL36**	mitochondrial ribosomal protein L36	0.77	0.76
**MRPL37**	mitochondrial ribosomal protein L37	0.77	0.73
**PUS1**	pseudouridylate synthase 1	0.73	0.75
**TSFM**	Ts translation elongation factor. mitochondrial	0.76	0.75

Mitochondrial protein import		#1	#2
**TIMM50**	translocase of inner mitochondrial membrane 50 homolog	0.64	0.62
**TIMM8B**	translocase of inner mitochondrial membrane 8 homolog B	0.76	0.80
**TOMM40**	translocase of outer mitochondrial membrane 40 homolog	0.69	0.77
**SAMM50**	sorting and assembly machinery component 50 homolog	0.54	0.69

Mitochondrial metabolites import		#1	#2
**SLC25A1**	solute carrier family 25 (mitochondrial citrate transporter) member 1	0.58	0.61
**SLC25A11**	solute carrier family 25 (mitochondrial oxoglutarate carrier) member 11	0.72	0.78
**SLC25A12**	solute carrier family 25 (mitochondrial carrier. Aralar) member 12	**3.21**	**1.31**
**SLC25A21**	solute carrier family 25 (mitochondrial oxodicarboxylate carrier) member 21	0.11	0.05
**SLC25A17**	solute carrier family 25 (mitochondrial carrier; peroxisomal membrane protein) member 17	0.62	0.47
**SLC25A37**	solute carrier family 25 member 37 (iron transport)	0.47	0.41
**SLC25A28**	solute carrier family 25 member 28 (iron transport)	0.78	0.75

Fatty acid metabolism		#1	#2
**AACS**	acetoacetyl-CoA synthetase	0.72	0.66
**ACAD9**	acyl-CoA dehydrogenase family. member 9	0.80	0.71
**ACSL3**	acyl-CoA synthetase long-chain family member 3	0.39	0.58
**ACSL5**	acyl-CoA synthetase long-chain family member 5	0.53	0.72
**ACSM3**	acyl-CoA synthetase medium-chain family member 3	**1.45**	**5.12**
**ACSS1**	acyl-CoA synthetase short-chain family member 1	0.58	0.62
**AUH**	AU RNA binding protein/enoyl-CoA hydratase	**1.40**	**1.52**
**FADS1**	fatty acid desaturase 1	0.61	0.63
**GPD1L**	glycerol-3-phosphate dehydrogenase 1-like	0.66	0.78

Other mitochondrial metabolic pathways		#1	#2
**ADCK2**	aarF domain containing kinase 2 (**CoQ10 synthesis**)	0.65	0.53
**COASY**	CoA synthase (**biosynthesis of Coenzyme A**)	0.71	0.76
**COQ3**	coenzyme Q3 homolog. methyltransferase (**CoQ10 synthesis**)	0.60	0.66
**DLD**	dihydrolipoamide dehydrogenase (**pyruvate dehydrogenase and TCA cycle**)	0.67	0.60
**DLST**	dihydrolipoamide S-succinyltransferase (**pyruvate dehydrogenase and TCA cycle**)	0.68	0.81
**FDPS**	farnesyl diphosphate synthase (**CoQ10 synthesis**)	0.68	0.63
**GOT2**	glutamic-oxaloacetic transaminase 2. mitochondrial (**aminoacids metabolism**)	0.67	0.80
**IDH2**	isocitrate dehydrogenase 2 (NADP+). mitochondrial (**TCA cycle**)	0.81	0.71
**OGFOD2**	2-oxoglutarate iron-dependent oxygenase domain 2 (**TCA cycle**)	0.71	0.70
**PDK3**	pyruvate dehydrogenase kinase. isozyme 3 (**pyruvate dehydrogenase**)	0.53	0.68
**PPCS**	phosphopantothenoylcysteine synthetase (**biosynthesis of Coenzyme A**)	**1.85**	**1.55**

Metabolism and transport of nucleotides		#1	#2
**DTYMK**	deoxythymidylate kinase (thymidylate kinase)	0.61	0.57
**DPYSL2**	dihydropyrimidinase-like 2	0.70	0.50
**GUCY1A3**	guanylate cyclase 1. soluble. alpha 3	**2.97**	**4.67**
**NT5DC2**	5-nucleotidase domain containing 2	0.68	0.58
**OGT**	O-linked N-acetylglucosamine (GlcNAc) transferase	**1.52**	**1.52**
**P2RY8**	purinergic receptor P2Y. G-protein coupled. 8	**1.49**	**2.30**
**PNPO**	pyridoxamine 5-phosphate oxidase	0.42	0.51
**POP7**	processing of precursor 7. ribonuclease P/MRP subunit	0.62	0.61
**PRPS1**	phosphoribosyl pyrophosphate synthetase 1	0.68	0.77
**PUS1**	pseudouridylate synthase 1	0.73	0.75
**RAVER1**	ribonucleoprotein. PTB-binding 1	0.58	0.55
**SLC29A1**	solute carrier family 29 (nucleoside transporters). member 1	0.55	0.72
**SLC29A2**	solute carrier family 29 (nucleoside transporters). member 2	0.68	0.77
**TK1**	thymidine kinase 1. soluble	0.78	0.46
**UGGT1**	UDP-glucose glycoprotein glucosyltransferase 1	0.68	0.64
**UMPS**	uridine monophosphate synthetase	0.49	0.55
**UXS1**	UDP-glucuronate decarboxylase 1	**1.51**	**2.07**
